# Somite Division and New Boundary Formation by Mechanical Strain

**DOI:** 10.1016/j.isci.2020.100976

**Published:** 2020-03-13

**Authors:** Ben K.A. Nelemans, Manuel Schmitz, Hannan Tahir, Roeland M.H. Merks, Theodoor H. Smit

**Affiliations:** 1 Department of Orthopaedic Surgery, Amsterdam University Medical Centres, Amsterdam Movement Sciences, Meibergdreef 9, 1105AZ Amsterdam, the Netherlands; 2 Centrum Wiskunde & Informatica, Science Park 123, 1098 XG Amsterdam, the Netherlands; 3 Mathematical Institute Leiden, Leiden University, Niels Bohrweg 1, 2333 CA Leiden, the Netherlands; 4 Department of Medical Biology, Amsterdam University Medical Centres, Meibergdreef 9, 1105AZ Amsterdam, the Netherlands

**Keywords:** Poultry Embryology, Mechanical Modeling, Developmental Biology

## Abstract

Somitogenesis, the primary segmentation of the vertebrate embryo, is associated with oscillating genes that interact with a wave of cell differentiation. The necessity of cell-matrix adherence and embryonic tension, however, suggests that mechanical cues are also involved. To explicitly investigate this, we applied surplus axial strain to live chick embryos. Despite substantial deformations, the embryos developed normally and somite formation rate was unaffected. Surprisingly, however, we observed slow cellular reorganizations of the most elongated somites into two or more well-shaped daughter somites. In what appeared to be a regular process of boundary formation, somites divided and fibronectin was deposited in between. Cell counts and morphology indicated that cells from the somitocoel underwent mesenchymal-epithelial transition; this was supported by a Cellular Potts model of somite division. Thus, although somitogenesis appeared to be extremely robust, we observed new boundary formation in existing somites and conclude that mechanical strain can be morphologically instructive.

## Highlights

• Live chick embryos develop normally under substantial axial strain (>50%)
• Mature somites divide into daughter somites, and fibronectin is deposited in between
• Mesenchymal cells from the somitocoel transition into epithelial border cells
• Mechanical strain can induce border formation and thus affect morphogenesis

## Graphical Abstract

**Figure:**
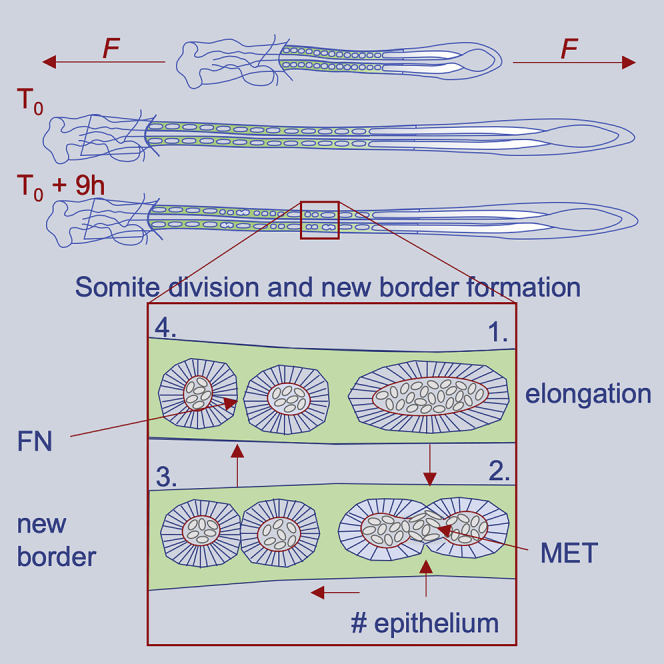

## Introduction

During morphogenesis, cells and tissues are subject to mechanical forces. These are generated by the cells themselves through cell-cell adhesion and contraction (Heer and Martin, 2017) and by external cues like osmotic pressure, growth, and forces generated by other cells. As cells are mechanosensitive, mechanical stress feeds back on cell behavior and can thus be considered instructive, e.g., as an organizing factor (Kumar et al., 2017) or as an activator of signaling pathways (Hubaud et al., 2017). This suggests that, alongside diffusible morphogens (Wolpert, 1969), mechanical strain is a provider of positional information (Miller and Davidson, 2013, Turing, 1952).

Somitogenesis involves the periodic organization of mesenchymal cells from the presomitic mesoderm (PSM) into cohesive clusters with an epithelial boundary. These clusters—somites—underlie the segmentation of the vertebrate body as they develop further into vertebrae and ribs, form the myotomes (the anlagen of the axial muscles), and impose segmentation on the peripheral nervous system. Somite formation is associated with genetic oscillations, which appear to be intrinsic to the cells of the PSM (Hubaud et al., 2017, Lauschke et al., 2012); this is described in the clock-and-wavefront model where molecular oscillators originating at the caudal end of the PSM (the clock) interact with a traveling front of maturation (the wave) created by antagonistic signaling gradients (Hubaud and Pourquié, 2014). The physical separation of a somite from the PSM correlates with the periodic expression of ephrin receptor A4 (EphA4) that interacts with ephrinB2 to induce cellular repulsion and cleft formation (Watanabe et al., 2009). This new boundary is then stabilized by the epithelization of the boundary cells (Martins et al., 2009) and the assembly of a fibronectin matrix in between (Rifes and Thorsteinsdóttir, 2012). Somite formation requires the condensation of cells in the PSM (Duess et al., 2013), the intercellular epithelial connection by N-cadherins (Horikawa et al., 1999), and cellular adhesion to the fibronectin surrounding the PSM (Georges-Labouesse et al., 1996, Martins et al., 2009). Somitogenesis is further facilitated by a tension on the embryo (Stern and Bellairs, 1984), which is naturally provided by the blastoderm that expands along the vitelline membrane (New, 1959). These studies indicate that not only molecular signaling, but also mechanical cues are involved in somite formation.

Considering then a possible role for mechanics in somitogenesis, we hypothesized that external mechanical strain might affect segmental patterning in the vertebrate embryo. Stern and Bellairs had inhibited the natural strain of live chick embryos by attaching them to a substrate; they observed a substantial widening of somites and eventually a secondary, lateral division (Stern and Bellairs, 1984). We applied a surplus longitudinal strain to the embryo, well beyond the natural tension of the blastoderm. We expected that such tension would induce a change in somite number or formation rate, but somitogenesis was essentially unaffected. Instead, and much to our surprise, the most elongated somites underwent slow subdivisions in what appeared to be a regular process of boundary formation, giving rise to what we designate as daughter somites. Here we report on these somite divisions and present a Cellular Potts model (CPM), which indicates that somite subdivisions may involve a mesenchymal to epithelial transition.

## Results

### Embryo Stretching Protocol

**Figure 1:**
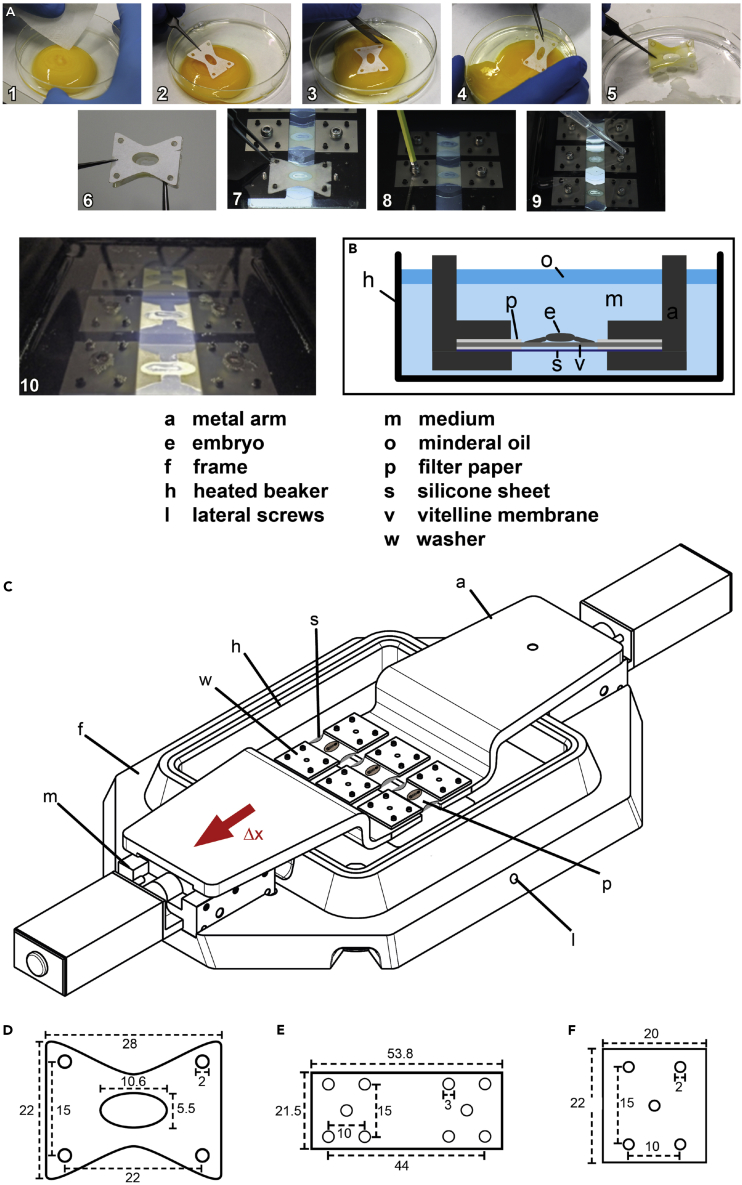
Chick Embryo Stretching *Ex Ovo*, Experimental Setup (A) Procedure of chick embryo explantation (Schmitz et al., 2016). (1) An egg is cracked into Petri dish and thick albumen removed from top of the yolk. (2) A filter paper carrier is placed on top of the yolk, surrounding the blastoderm and a substantial area of the vitelline membrane. (3) The filter paper carrier is cut loose from the surrounding vitelline membrane and (4) removed from top of the yolk. (5) Remaining yolk is carefully washed away in a saline bath. (6) The embryo is sandwiched with a second filter paper carrier. (7) The filter paper sandwich is submerged into the medium and hooked into the pins of the motorized arms. A thin sheet of PDMS below the embryo and vitelline membrane protects the embryo from convection of the medium. (8) Washer plates clamp the filter paper sandwich to the metal arms and are carefully pressed down by nuts. (9) Filter paper sandwiches are cut perpendicularly at mid-level of the embryo for later stretching of embryos and the medium is covered with a layer of light mineral oil to prevent evaporation. (10) Three embryo sandwiches are pulled simultaneously under microscopic imaging to create time-lapses. (B) Schematic cross-sectional view of the chick embryo (E) mounted along with the vitelline membrane (v) in a filter paper sandwich (p). The entire sandwich is supported by a flexible sheet of PDMS (s) and attached to the metal arms (A). The embryo is submerged in medium (m) in a heated beaker (H). A layer of mineral oil (o) prevents the medium from evaporating. (C) Schematic view of the embryo stretcher. The frame carries the motorized stages and keeps the temperature-controlled medium container in position. The whole setup is placed on a motorized x-y-stage, embryos are imaged from above and illuminated from below through the glass bottom of the medium container. (D) Filter paper carrier dimensions (in mm). (E) Dimensions of stencil for PDMS sheets (in mm). (F) Dimensions of metal washers used to clamp the filter paper (in mm).

Stage HH8-9 chick embryos were cultured *ex ovo* in modified submerged filter paper sandwiches (Figure 1) (Schmitz et al., 2016). They were stretched along their body axis in the filter paper sandwiches at a continuous rate of 8 μm/min, resulting in an elongation of the embryo of about 4 μm/min. After 16 h of elongation, we observed the division of somites for the first time. However, in this stretching protocol many of the embryo cultures were torn by the excessive tension, which impeded repeatability. To optimize the stretching protocol, we strained the embryos in two sessions of 51–55 min at 1.2 μm/s, separated by a resting period of 2 h, during which the samples could relax and repair (Figure S1). This protocol resulted in a sample elongation of 7.6 mm, equaling the 16 h of continuous stretching at 8 μm/min. The embryos themselves experienced strains of 23 ± 3% (average ±SD; n = 57) during the first pull and 19 ± 3% during the second pull, on top of the natural growth of the embryo and viscous relaxation (Figure S1). The total elongation of the embryos was around 70%–80% for the experimental group and 25%–30% for the controls (Figure S1). The variations in strain are due to variability in original embryo length and biological variation in stiffness of both embryo and the supporting membrane.

### Somitogenesis Is Normal, but Somites Divide

**Figure 2:**
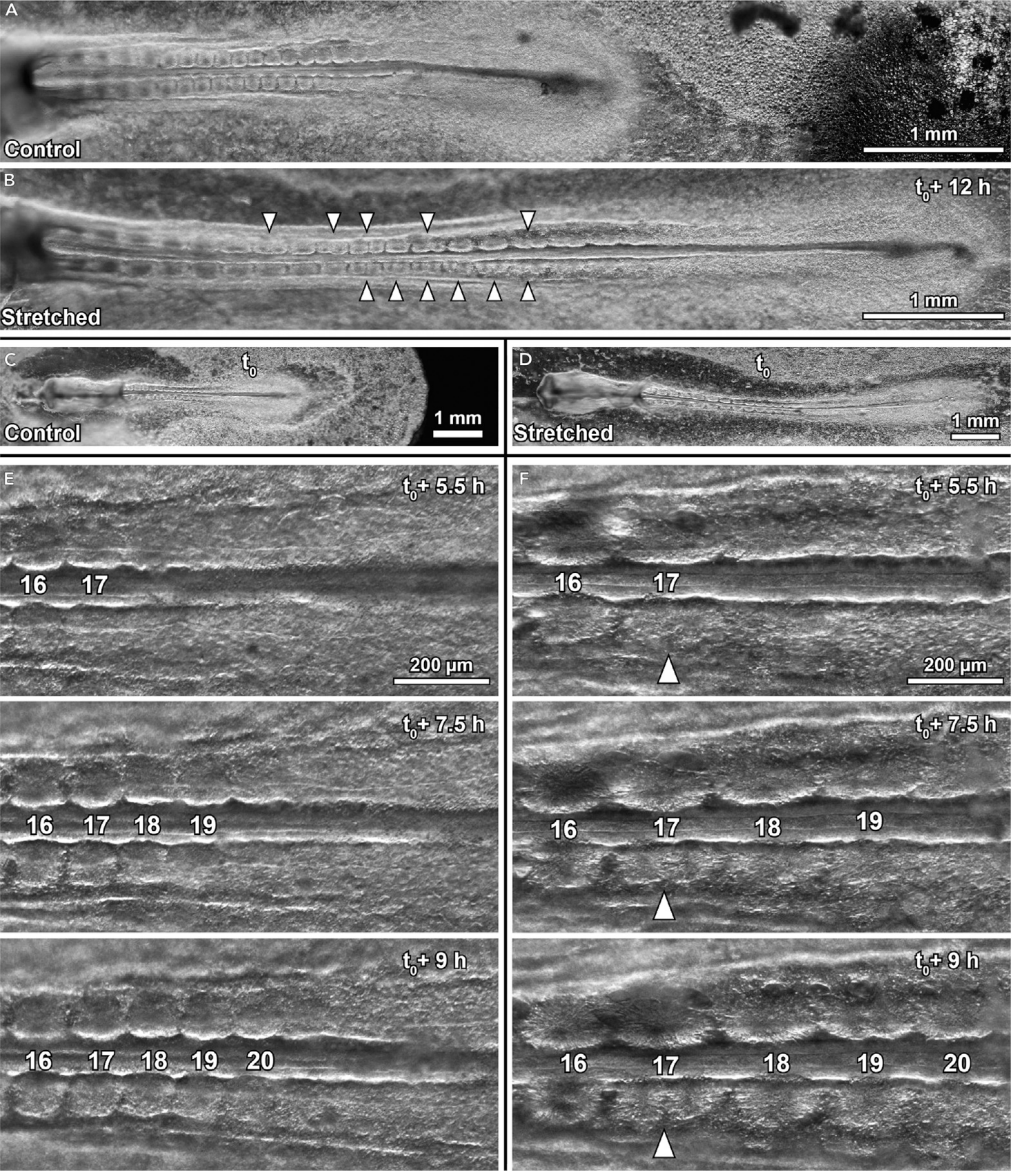
Daughter somite Formation in Stretched Chicken Embryos Dark-field microscopy images of age-matched control (A) and stretched embryo (B). Anterior is to the left in all images, white arrowheads indicate daughter somite formation; t_0_ marks the end of the stretching protocol (Figure S1), ventral view. Difference in axial length becomes obvious between control embryo (C) and stretched embryo (D) (at t_0_ both embryos are at the 13-somite stage). Selected time-lapse frames of the segmenting PSM in control (E) and stretched embryo (F). The numbers in (E) and (F) indicate the total number of somites in the embryo, not the identity of the specific somites.

After the second pull (t_0_), we monitored the strained embryos for another 12 h. Time-lapse microscopic imaging revealed that somites budded off from the PSM with the same period in stretched samples (80 ± 6 min/somite) as in controls (79 ± 8 min/somite), but as expected the stretched somites were more elongated (Figure 2; Videos S1 and S2). Strikingly, however, the most deformed somites then divided into what we call “daughter somites” (Figure 2). During this process, the deformed mother somites invaginated along the medio-lateral plane (Figures 2E and 2F). This occurred simultaneously to or after their separation from the PSM and took more than 5 h (about four somite periods) from the first appearance of an invagination to complete boundary formation between the daughter somites.

Video S1. Control Embryo, Related to Figures 2A, 2C, and 2E Time-lapse Video of the development of a control chick embryo (stage HH9+), cultured ex ovo, mounted in submerged filter paper sandwiches in the stretch setup for 19 h, without stretching. Timer is in hours.

Video S2. Stretched Embryo Showing Somite Divisions, Related to Figures 2B, 2D, and 2F Time-lapse Video of the development of an experimental chick embryo (stage HH10), cultured *ex ovo*, mounted in submerged filter paper sandwiches in the stretch setup for 17.5 h, and stretched at a speed of 1.2 μm/s along the anterior-posterior (AP) axis, in two pulls. First the overview is shown, afterward a zoom in at the mesoderm. The stretching deforms the embryos slowly but substantially, while development progresses without damage. During the deformation, somites divide into daughter somites of different sizes, as marked by the white arrowheads in the zoom. For example: the first arrowhead shows an asymmetric somite division (lower left), whereas the second arrowhead shows a symmetric division (upper left).

**Figure 3:**
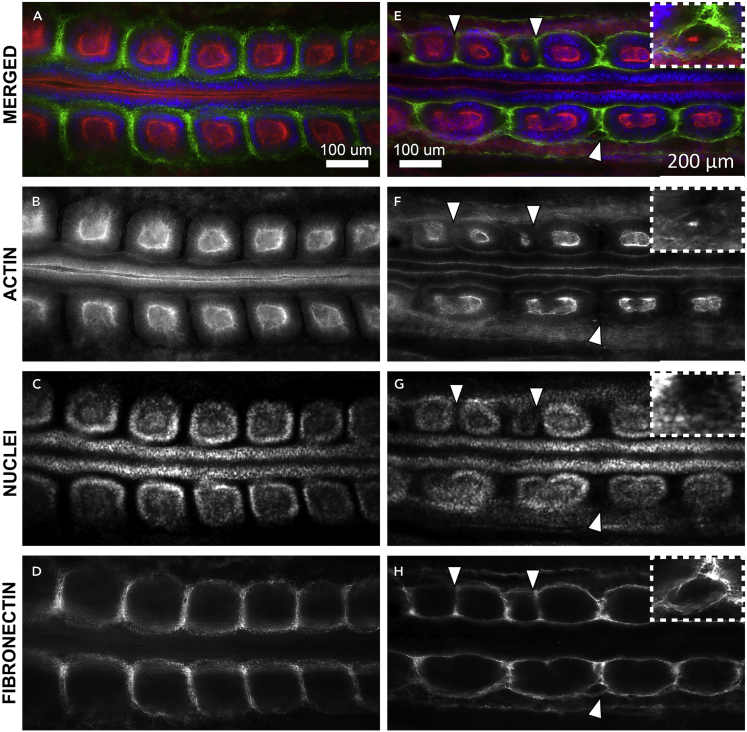
Fibronectin Distribution Around Daughter Somites Widefield fluorescent micrographs of control embryo (A–D) and stretched embryo (E–H) stained for actin (red), DNA in cell nuclei (blue), and extracellular matrix component fibronectin (green). Ventral view, anterior is to the left. Daughter somites are surrounded and separated from each other by a newly formed fibronectin matrix (white arrowheads in [E] and [H]) and can be extremely small (inset [E]–[H]) and lacking a mesenchymal core. Photo credit: Ben Nelemans, Amsterdam University Medical Centres.

Somite division in stretched embryos appeared unilaterally or bilaterally and often resulted in daughter somites of different sizes (Figures S2 and S3). Daughter somites consisting of only a few epithelial cells were also observed (Figure 3E).

### Daughter Somites Form New Epithelial Boundaries

**Figure 4:**
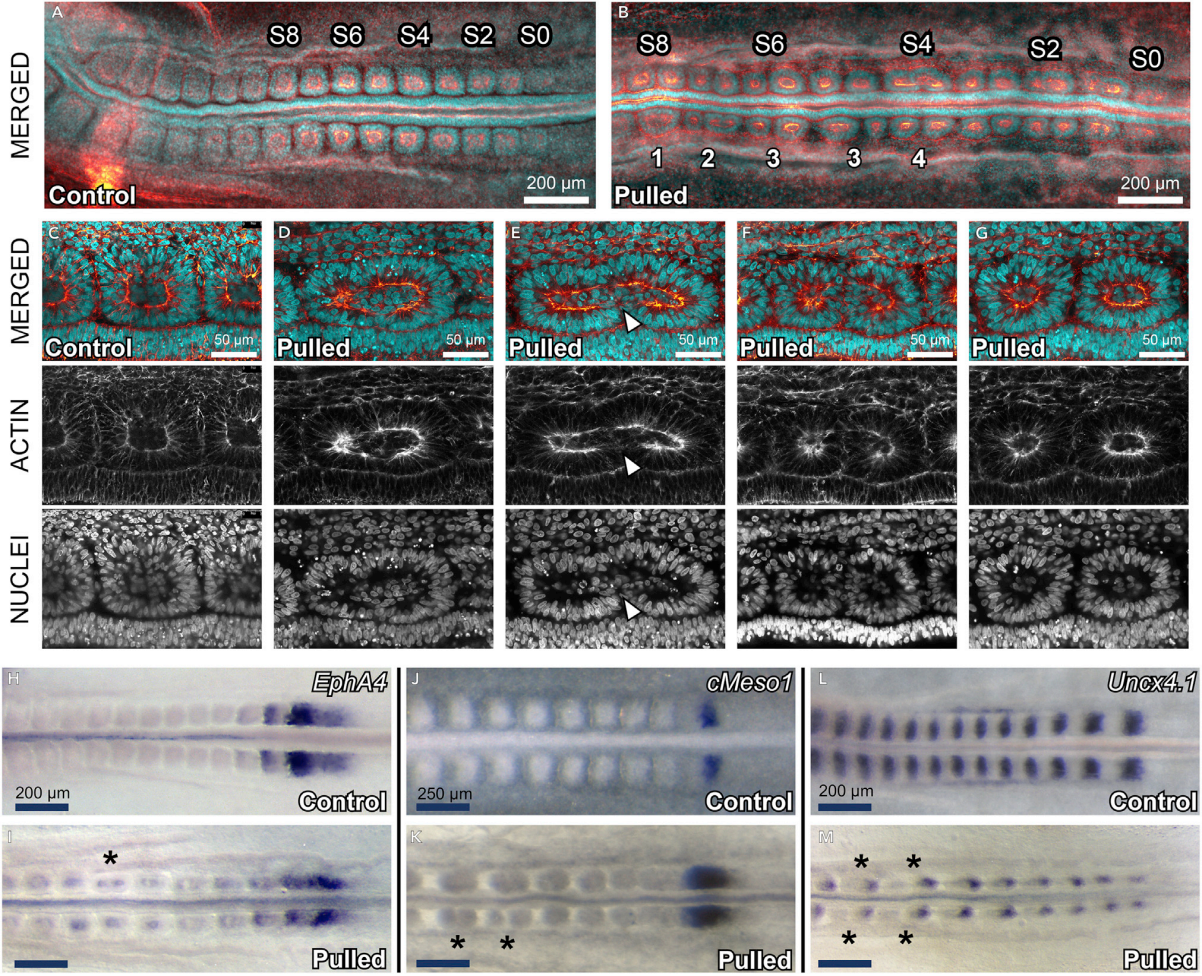
Immunohistochemistry and *In Situ* Hybridizations of the Embryos (A and B) Control (A) and stretched embryo (B), anterior is left, somite numbers are indicated (Christ and Ordahl, 1995). Scale bars, 200 μm. (B) Daughter somites can form unilaterally (1), equally (4), or unequally sized (3) and subdivide further (2). (C–G) Confocal cross sections of selected somites of the same control (C) and stretched embryo (D–G). Anterior is left and medial below. Panels are arranged in potential order to illustrate the transition from a mechanically deformed somite (D) to two daughter somites (G). Cells from the somitocoel seem to be incorporated into the epithelium at the site where the epithelium is ruptured (arrowhead in [E]). (H–M) *In situ* hybridizations for *EphA4*, *cMeso1*, and *Uncx4*.*1* show that *EphA4* expression is maintained or induced around the somitocoels (I), whereas no new rostro-caudal polarity is induced in the daughter somites (indicated by ∗).

We fixed and immunohistochemically stained the stretched embryos (Figures 3 and 4). Under wide-field microscopy, daughter somites appeared as stable, clearly separated cellular spheres enclosed by a fibrous extracellular matrix (ECM) staining positive for fibronectin (Figure 3, 4A, and 4B). Somites in control embryos were round (Figures 3A–3D), whereas those in stretched embryos were strongly deformed to an elliptical shape, with the epithelial cells organized radially around a somitocoel of mesenchymal cells (Figures 3E–3H). We identified potential transitional stages of daughter somite formation (Figures 4C–4G). The apical actin cortices of these somites showed discontinuities along their mediolateral planes, indicating openings of the epithelial sheet under mechanical strain (Figure S4). At these locations, mesenchymal cells from the somitocoel may integrate into the existing epithelium.

### Mechanical Strain Appears to Activate EphA4, but Not Uncx4.1 or cMeso1

To determine whether mechanical strain re-activated genes related to somitic boundary formation, we stained for *EphA4* mRNA expression (Figures 4H and 4I). EphA4 is reported to induce somite detachment through the repulsion of ephrinb2 (Watanabe et al., 2009). *EphA4* was consistently expressed ectopically at the apical side of the epithelium of the stretched somites (Figure 4I), albeit at a much lower level than at somite 0 in both stretched and control somites (Figures 4H and 4I). There was no *EphA4* expression in the somites of the unstrained controls older than somite I (Figure 4H). Stretching did not affect the expression of *cMeso1* (Figures 4J and 4K), the key initiator of somite rostro-caudal polarity in chicken (Morimoto et al., 2007), or the expression of the caudal somite marker *Uncx4*.*1* (Schrägle et al., 2004) (Figures 4L and 4M); this indicates that the clock-and-wavefront mechanism is operating normally in stretched embryos.

### Cellular Potts Model of Somite Division

In order to obtain a better understanding of the cellular reorganization during somite division, we developed a cell-based, two-dimensional CPM, implemented in the open-source package CompuCell3D (Glazier and Graner, 1993). The default hypothesis for the splitting of rod-shaped clusters of cohesive cells into a series of spherical aggregates is a mechanism known as the Plateau-Rayleigh instability (Hutson et al., 2008). This mechanism is only possible in three dimensions, because in two dimensions there is no ring tension. Using viscous liquid models of tissue mechanics, Grima and Schnell argued that for typical values of the tissue surface tension and bulk viscosity of embryonic tissues, such surface-tension-driven mechanisms are likely not fast and strong enough to be the main driving forces of somite formation Grima and Schnell, 2007. Therefore, we turned to a more complex model, which we simulated in 2D for computational efficiency.

**Figure 5:**
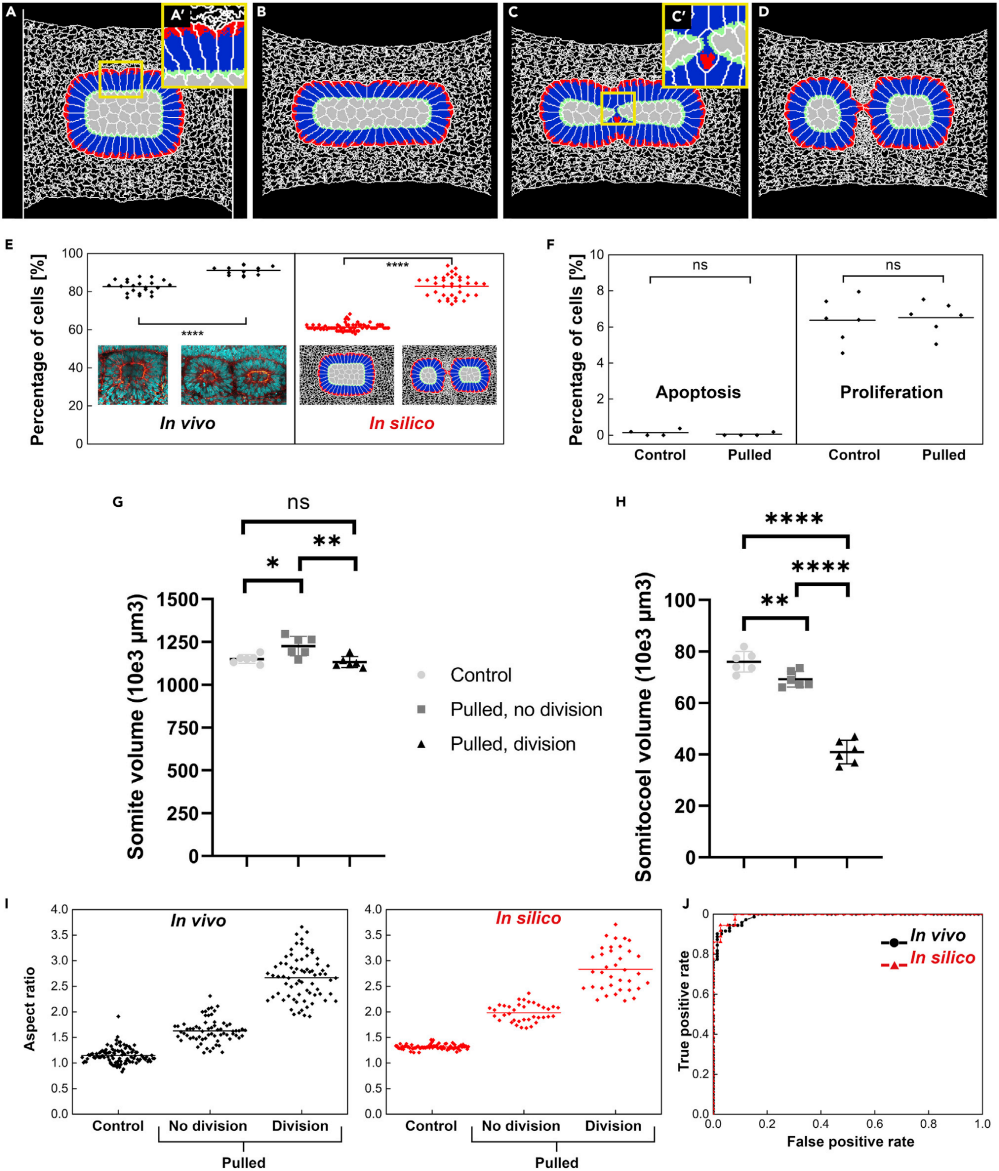
Cellular Potts Model of Somite Remodeling Compared with *In Vivo* (A–D) Daughter somite formation *in silico*, induced by stretching. Mesenchymal cells (gray) and extracellular matrix (white mesh), (A′) epithelial cells consisting of apical (green), lateral (blue) and basal (red) domains. (C′) Somitocoel cells undergoing MET. (E) There is a significant increase of epithelial cell fraction *in vivo* (p < 0.0001) and *in silico* (p < 0.0001). (F) Apoptotic and proliferation rates in somitic mesoderm of control and stretched embryos; differences are non-significant (Mann-Whitney test, apoptosis p = 0.43, proliferation p = 0.79). (G) Volumes of control (unstrained), strained, and daughter somites S5 and S6. Volumes of divided somites are summed from two daughter somites and show no significant volume difference to control somites. There is a temporary increase in volume, when somites are pulled, but have not yet divided. ns, not significant; ∗: p<0.05; ∗∗: p<0.01 (H) Somitocoel volumes in the same somites, showing a strong decrease of mesenchymal volume after somite splitting (p < 0.0001), thereby confirming mesenchymal-epithelial transition. ∗∗: p<0.01; ∗∗∗: p<0.001; ∗∗∗∗: p<0.0001. (I) Somite aspect ratios for controls and non-divided and divided somites *in vivo* and *in silico*. (J) ROC curves for daughter somite formation *in vivo* and *in silico*, in dependence of aspect ratio of deformed somites. AUC is 0.989 (*in vivo*, 95% confidence interval [CI]: 0.953–0.999) and 0.993 (*in silico*, 95% CI: 0.938–1.000). Dots in Figures 5E–5I are individual data points, the lines indicating their mean values. Figures 5G and 5H also include the Standard Error of the Mean (SEM).

We initialized our simulations with a somite consisting of a core of non-polarized mesenchymal cells surrounded by a layer of polarized, epithelial cells (Dias et al., 2014), embedded in an elastic extracellular matrix (ECM; Figure 5A). We mimicked stretching by applying axial tension to the ECM (Video S3). Epithelial cells are mutually coupled by tight junctions, linked intracellularly to the cytoskeleton (Figure S5) (Kim et al., 2017). Elastic springs coupled the apical sides of the simulated epithelial cells to one another, whereas a set of intracellular springs represented the cytoskeleton (Figure S5) (Dias et al., 2014).

Video S3. Somite Epithelialization and Division *In Silico*, Related to Figure 5 Simulation video of dividing *in silico* somites, consisting out of frames of every 500^th^ Monte Carlo Step, starting at briefly before pulling at 60,000 MCS (∼6 h), and ending at 130,000 MCS (∼13 h). Among alternative, unsuccessful mechanisms (Figure S6) daughter somite formation in stretched somites could be reproduced if we assumed MET was induced in mesenchymal core cells upon contact to the basal or lateral membranes of epithelial cells.

### MET May Occur through Lateral Induction of Broken Epithelium

First, we tested if stretching and reorganization of the mesenchymal and epithelial cells sufficed for somite division. However, none of the strains tested induced somite division (Figure S6, top row). Considering then that two daughter somites need more epithelial cells than one mother somite, we assumed that the mesenchymal cells from the somitocoel could transition into the epithelium, thus mimicking a mesenchymal-epithelial transition (MET). We tested two rules for inducing MET in the deformed somite. In the first scenario, a mesenchymal cell underwent MET after full migration into the epithelial layer and contact with the surrounding ECM for 600 time steps (counted in Monte Carlo Steps [MCS], see Methods), corresponding to approximately 4 min (see Table S1). We did not observe somite division for different levels of deformation, possibly because there was insufficient contact between mesenchymal somitocoel cells and the surrounding ECM (Figure S6, middle row). In the second scenario, mesenchymal cells underwent MET after extended (600 MCS or 4 min) contact with the basal or lateral membrane of the epithelial cells. Upon stretching, the connections between the epithelial cells snapped and the mesenchymal cells from the core became exposed to their lateral or basal membranes. This induced MET and the cells were integrated into the epithelial layer (Figure S6, lower row; Video S3). We also observed that a decreased cohesion between the lateral domains of epithelial cells resulted in subdivisions into small cell clusters of epithelioid morphology (Figure S7), similar to the subsomites observed in *N-Cadherin/cad11* double-homozygous mouse mutants (Horikawa et al., 1999).

### Ratio of Mesenchymal/Epithelial Cells Decreased

Integration of the mesenchymal cells into the epithelial layer inherently increases the somite's surface-volume ratio. Also *in vivo* the epithelial cell fraction was significantly higher in daughter somites than in controls and matched the *in silico* prediction (Figure 5E). Importantly, the stretching *in vivo* caused no significant changes in apoptosis (p = 0.4286) or proliferation rates (p = 0.7879) (Figure 5F). We also compared the volumes of somites and somitocoels between control, stretched, and divided somites V and VI (Figures 5G and 5H). These quantifications show that stretched somites increase in volume before division. After division, however, the total volume of the daughter somites does not significantly differ from control somites, suggesting again that the same number of cells are kept during the cellular rearrangement and that the cell ratio is not related to any volumetric effect (Figure 5G). Finally, divided somitocoels show a ∼50% smaller volume compared with control or stretched somites (Figure 5H). Altogether, the data suggest that MET is the most likely explanation for the observed increase in epithelial cell fraction.

### Mechanical Deformation beyond Threshold Induces Somite Division

To determine the relationship between mechanical deformation of somites and the probability of somite division, we compared the aspect ratios of stretched dividing somites (prior to division) and stretched non-dividing somites with those of control somites (Figure 5I). These measurements show that somite division only occurs beyond an aspect ratio threshold of approximately 2.0 *in vivo* or 2.5 *in silico*. The corresponding receiver operating characteristics (ROC) curves (Hanley and McNeil, 1982) show that somite aspect ratio is indeed an excellent predictor of somite division, both *in vivo* and *in silico* (Figure 5J).

## Discussion

Vertebrates are characterized by their segmented body plan, first visible in the somites that form along the embryonic body axis. The numbers of somites and vertebrae are remarkably constant within species, although genetic mutations can slightly alter somite number and formation period (Schröter and Oates, 2010). Considering that morphogenesis is also a physical process, we hypothesized that mechanical strain might affect both the formation rate and the number of somites. In order to test this, we subjected chick embryos to a substantial mechanical strain, resulting in an elongation more than twice the natural lengthening of an embryo. Based on the stiffness (2.4 kPa) and size (84 by 200 μm) of the midline (notochord, neural tube, and somites) (Agero et al., 2010), a force of 8.4 μN is required to apply a strain of 23%. The stress applied on the midline after two pulls is about 1,200 Pa, well above the estimated yield stress in the anterior PSM (20–220 Pa) (Mongera et al., 2018) and apparently sufficient to break epithelial cell-cell adhesions.

Although the global forces applied are clearly supra-physiological, the average somite formation period remained essentially the same at approximately 80 min/somite. This shows that the clock-and-wavefront mechanism is extremely robust and not disturbed even by excessive mechanical strain. Surprisingly, however, beyond a threshold of somite deformation there was a slow reorganization of somites into two or more well-shaped daughter somites. Each division took about 6 h, similar to the period from the determination front (somite -IV) to the formation of somite I (Maroto et al., 2012). This indicates that daughter-somite formation is an active process of boundary formation, rather than an acute mechanical disruption.

Somite division starts with snapping of the somite epithelium, and one may wonder whether the process that follows is damage repair or normal morphogenesis. This is difficult to determine, because healing of biological tissues generally involves many processes that also occur during development. Furthermore, it is conceivable and in fact suggested (Bard, 1988, Truskinovsky et al., 2014) that local strains in the mesoderm may be several times the global embryo deformation due to differential straining. Indeed, the overstretching of a somite appears to create a situation that results in a morphogenetic process of new border formation. In this context it is interesting to note that the surplus strain applied to the live embryos affects cohesive (epithelial) cells in the anterior mesoderm (i.e., the somites), rather than the loose, granular (mesenchymal) cells in the PSM. This is commensurate with observations that cohesive granular materials crack under stretching, whereas dry, non-cohesive granules do not (Alarcón et al., 2010). It may also explain why somitogenesis, which essentially occurs in the mesenchymal PSM, is robust under supra-physiological strain and indeed may be insensitive to it.

Our mathematical modeling and microscopic observations suggest that the daughter somites are essentially composed of cells from the mother somite. This is confirmed by the observation that proliferation and apoptosis did not change under mechanical stretching. It further implies that the larger number of epithelial cells required to meet the demand of more border cells is met by mesenchymal-epithelial transition (MET). Both *in vivo* and *in silico* we observed that mechanical strain ruptured the apical actin cortices. The mesenchymal cells from the somite core come into contact with the lateral sides of the epithelial cells and presumably undergo mesenchymal-epithelial transitions (MET) (Jackson et al., 2017) to be integrated into the somitic epithelium. The ability of mesenchymal cells to revert to an epithelial identity demonstrates cell plasticity as suggested earlier (Dongre and Weinberg, 2019, Yang and Weinberg, 2008); our observations of somite divisions shows that interconversion between epithelial and mesenchymal cell states may also occur under mechanical conditions.

*In situ* hybridization for the Mesp2 homolog, Meso1, indicates that the clock-and-wavefront mechanism is operating normally in stretched embryos, presumably because mechanical strain does not affect loose, granular tissues like the mesenchyme of the PSM (Alarcón et al., 2010). We further consistently observed light ectopic expression of *EphA4* without c*Meso1* expression in the strained somites, although *EphA4* is thought to be downstream of c*Meso1* (Watanabe et al., 2009). Thus, somite division occurs without Meso1 expression defining the somitic border, as normally occurs during somitogenesis. This would indicate that *EphA4* expression in stretched somites is either maintained or reinitiated and suggests an alternative mechanosensitive pathway leading to EphA4 upregulation, independent from, or redundant to, c*Meso1*. However, we are hesitant to draw such firm conclusions, because the level of *EphA4* expression is much lower than observed in somite 0 in both the experimental and the control somites.

In 1984, Stern and Bellairs cultured chick embryos on agar-glucose-saline-albumen substrates, which in several cases inhibited their elongation and resulted in a PSM wider than normal (Stern and Bellairs, 1984). This PSM segmented in a normal rostral-caudal sequence, but the laterally elongated somites then subdivided secondarily into daughter somites of about normal size, that is, perpendicular to the direction of the maturation gradients. This indicates that boundary formation is induced independent from any clock-and-wavefront mechanism. It further appears that “wide” somites formed under compression are unstable, commensurate to the “long” somites produced under tension in the current study. We observed that the epithelial layer of the elongated somite ruptured and induced mesenchymal-epithelial transition of the somitocoel cells (Jackson et al., 2017). Whether the epithelial layer of the wide somites in Stern and Bellairs' study also ruptured under axial compression cannot be determined from the figures in the publication, but considering their strong lateral elongation with estimated aspect ratios well above 2.0, this is quite conceivable.

Daughter somite formation, both under tension and compression, suggest that boundary formation can take place outside the somite determination front (Hubaud and Pourquié, 2014), presumably in response to mechanical cues, and thus independently from the clock-and-wavefront mechanism. This was also the case with the extra-embryonic *de novo* formation of somites (Dias et al., 2014): like normal somites, these ectopic somites had epithelial layers surrounding mesenchymal cells and were embedded in a matrix of fibronectin, which is known to be an essential condition for somite formation (Rifes and Thorsteinsdóttir, 2012). Unlike the situation in the compressed or elongated embryos, however, there were no geometrical boundary conditions for the ectopic somites imposed by the surrounding structures, which allowed the reported unrestricted, grape-like somite formation in all directions. An alternative explanation for the somite division reported here and the ectopic somite formation reported by Dias et al. (2014) is offered by Horikawa and colleagues, who created N-Cadherin mutations in chick embryos and observed small, irregular somites, which they called *subsomites* (Horikawa et al., 1999). We used our CPM to investigate the role of cadherins *in silico* and found that reduced intercellular adhesion indeed results in small, irregular subsomites (Figure S7). In our experimental study, however, daughter somites were not created by reducing cellular adherence but by mechanical overstraining of the epithelial border of the somites; the feasibility of this mechanism was confirmed by the CPM (Figure 5). The mechanism of ectopic somite formation (Dias et al., 2014) is as yet less clear and in fact out of scope of the current study. Based on the current study we conclude that mechanical strain can induce border formation and thus control morphogenesis; this is an important concept to keep in mind when studying not only embryonic development, but also tissue homeostasis and disease.

### Limitations of the Study

Our study involves some limitations, which may be addressed in future studies. First, the straining protocol of two supra-physiological pulls, separated by 2 h of relaxation, looks quite arbitrary. Although we feel that a minimum of axial stretching is required to deform the somites and induce division and MET, other stretching regimes may have worked as well or even better. Alternatively, one may think about other (more physiological) ways to enhance differential strain between the rupturing epithelium and the surrounding tissues, connected to each other by fibronectin, e.g., by enhancing epithelial contraction.

Although our observations on border formation are robust and consistent, the case for MET would have been stronger if MET could have been shown in single cells. One way of doing that would be monitoring single mesenchymal cells of the somitocoel throughout the process of somite division, e.g., through DiI labeling of mesenchymal cells prior to stretching (Kulesa and Fraser, 2002). Alternatively, immunohistochemistry of single somites could highlight specific markers of MET or the epithelium. In our experimental setup this was technically not feasible because of the large, and somewhat unpredictable, displacement of somites prior to and during the stretching protocol.

## Methods

All methods can be found in the accompanying Transparent Methods supplemental file.

## Data and Code Availability

All data needed to evaluate the conclusions in the paper are present in the paper and/or the Transparent Methods. All data and the computational modeling code are available from authors upon request.

## Supplemental Information

Document S1. Transparent Methods, Figures S1–S9, and Tables S1
