# Supplementary material for: Somite Division and New Boundary Formation by Mechanical Strain
Source: iScience. 2020 Mar 13;23(4):100976. doi: 10.1016/j.isci.2020.100976 (PMC7109633; doi:10.1016/j.isci.2020.100976)
Supplement: Document S1. Transparent Methods, Figures S1–S9, and Tables S1 [file mmc1.pdf]

iScience, Volume *23*

Supplemental Information

Somite Division and New Boundary

Formation by Mechanical Strain

Ben K.A. Nelemans, Manuel Schmitz, Hannan Tahir, Roeland M.H. Merks, and Theodoor H. Smit

Supplemental Information

Supplemental figures

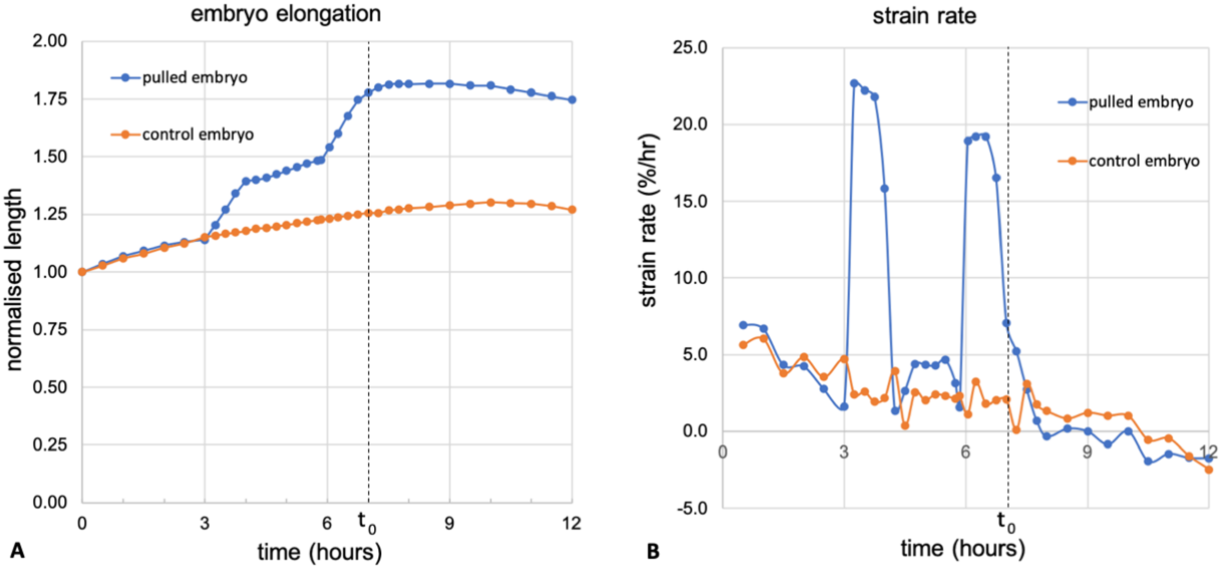

Fig S1: Natural and experimental elongation of chick embryos, related to figures 1 and 2, and Movies S1 and S2.
Left: embryo elongation normalized to the length at T=0. Right: strain rate (%/hr) for the same embryos. Orange: natural growth of the chick embryo is more than 25% after 9 hours. Elongation gradually slows down as embryo reaches the borders of the framed membrane (see also negative slope right). Blue: embryo pulled about 23% at T=3h, two hours rest, and again for 19% just before T=6h. The post-stretch period (t_0, see Fig.2) starts at T=7h. Total elongation including growth reaches 80% at T=8h. Note that the applied stretch does not affect the underlying elongation rate due to growth (negative slope similar to control embryo).

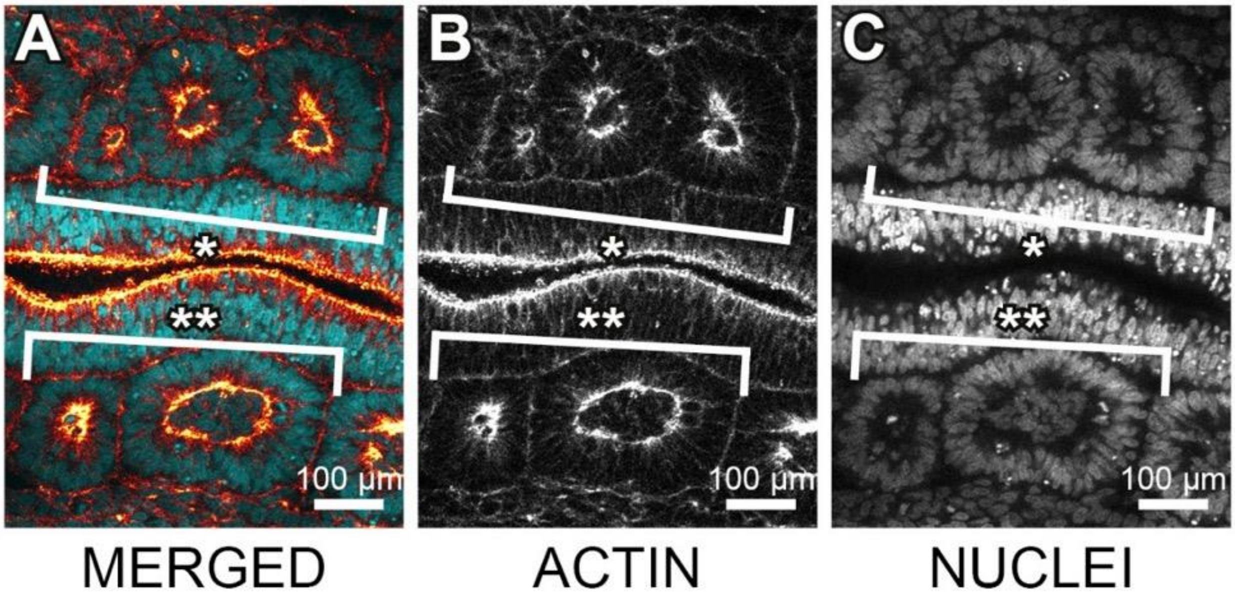

Fig S2: Versatile daughter somite morphologies, related to Figure 3. Confocal micrograph of daughter somites on both sides of the midline in stretched and fixated chick embryo stained for actin (red) and DNA in cell nuclei (blue). Anterior is to the left, ventral view. Daughter somites do not only result from splitting into anterior and posterior compartment of original somite, but can also reorganize into three daughter somites (*) or two unequally sized daughter somites (**).

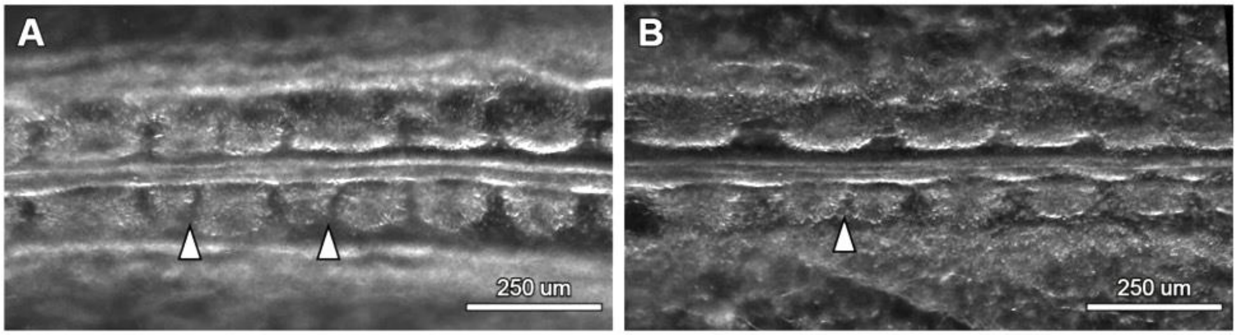

Fig S3: Unequally sized daughter somites, related to Figure 3. Anterior is to the left. White arrowheads indicate gaps between unequal daughter somite pairs.

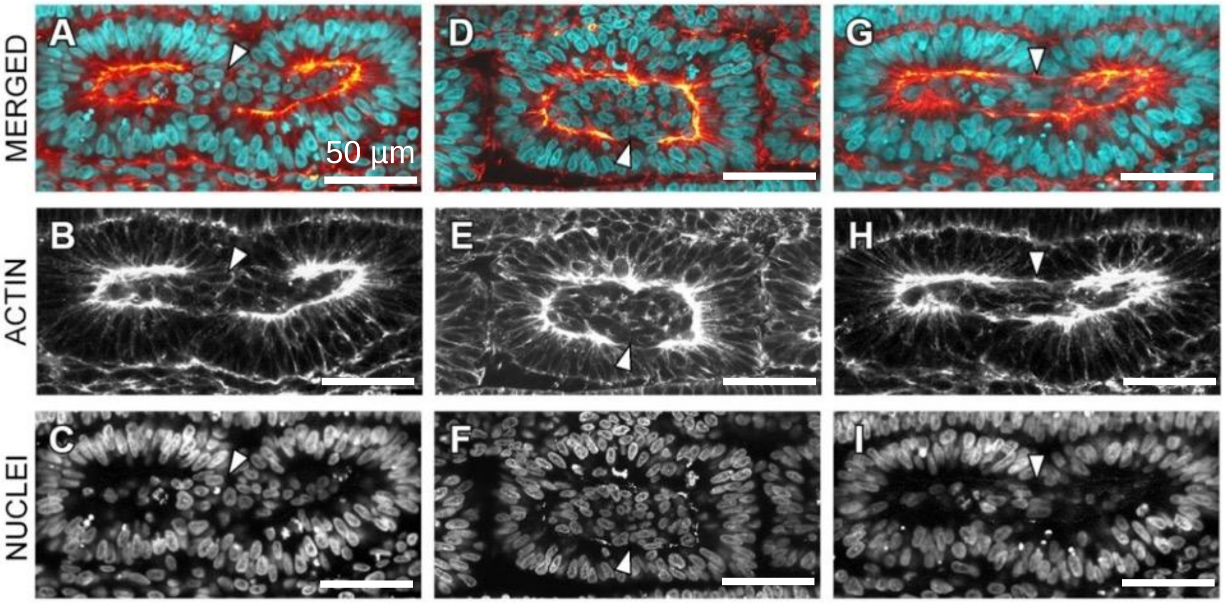

Fig S4: Fracture of epithelial sheet and potential MET of mesenchymal somitocoel cells, related to Figure 4. Confocal micrograph of somites, fixated during daughter somite formation in stretched chick embryos, stained for actin (orange) and DNA in cell nuclei (cyan). Anterior is to the left. White arrowheads indicate discontinuities in the apical actin ring of the somitic epithelium, suggesting a local opening of the epithelial sheet and potential interfaces for the recruitment of additional mesenchymal cells from the somitocoel for their incorporation into the existing epithelium.

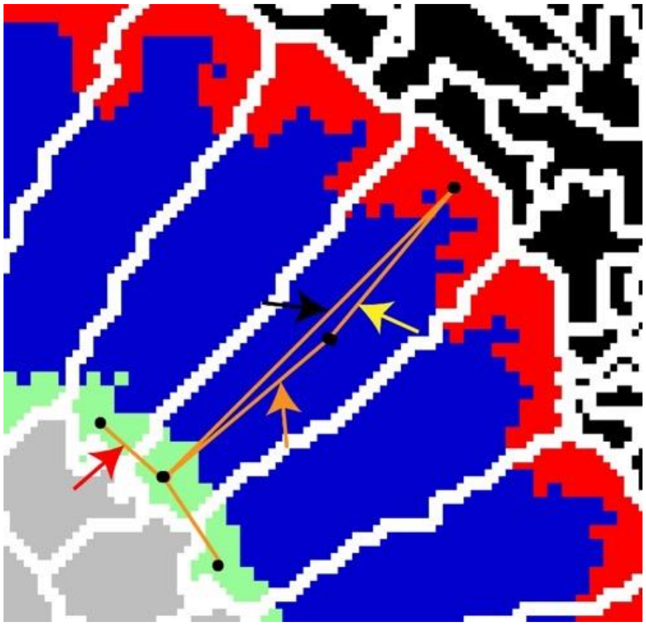

Fig S5: Snapshot of various cell types present in the simulations, related to Figure 5. Mesenchymal cells shown in grey. Epithelial cells consist of three domains: Apical (green), lateral (blue) and basal (red) domain. Centre of masses (black dots) of epithelial cell domains are connected internally to each other using elastic springs. The springs between apical and lateral domain (orange arrow), lateral and basal domain (yellow arrow) and apical and basal domain (black arrow) are indicated. Each epithelial cell has the same spring configuration helping epithelial cells to elongate after polarization. Apical domains of neighbouring are also connected via springs (red arrow) to trigger the formation of single layer of epithelial cells. Black cells in the upper right-hand corner represent ECM.

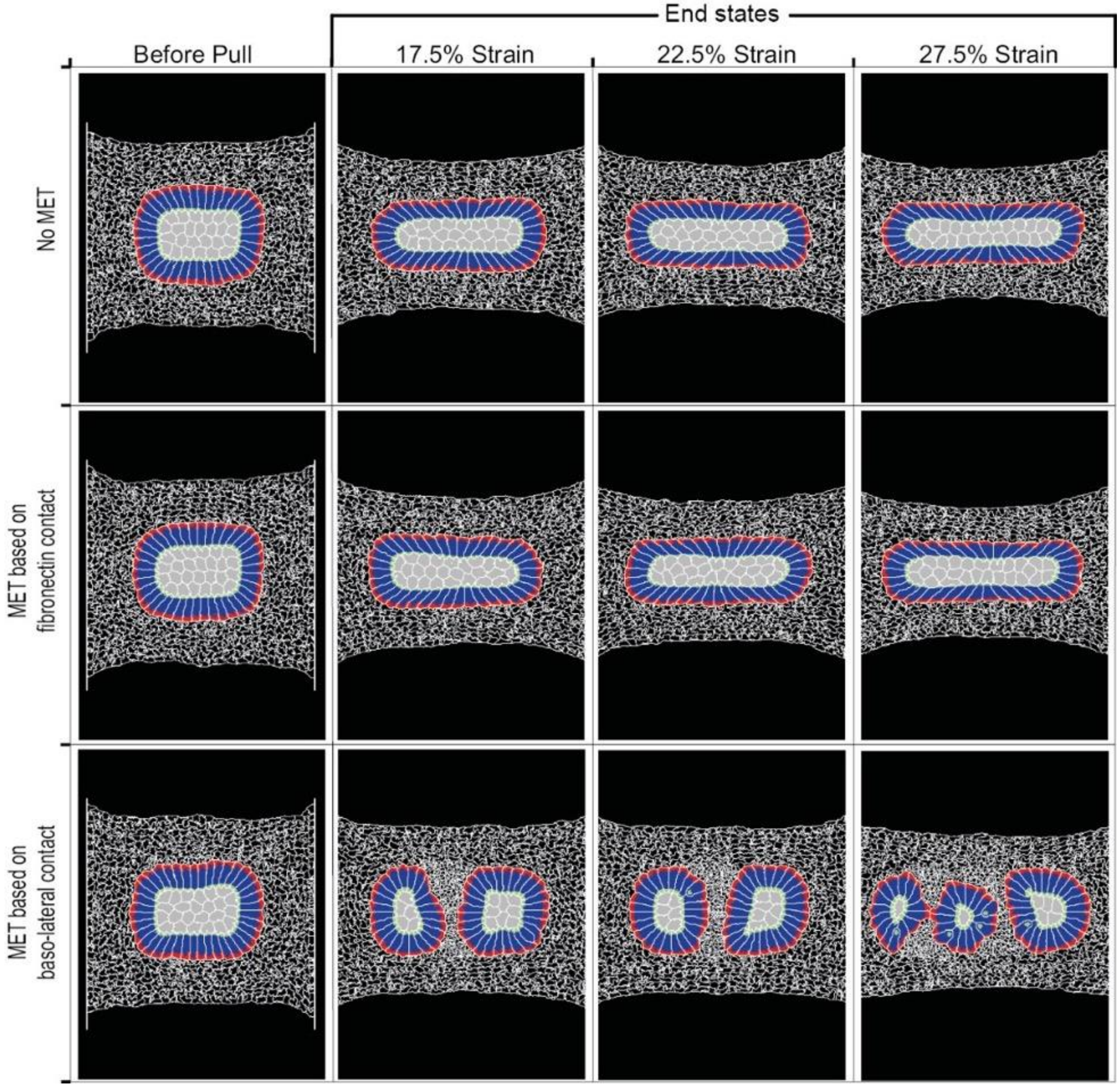

Fig S6: End states of simulations, testing different mesenchymal-epithelialization transition rules, related to Figure 5. Fully epithelialized somites were exposed to different strains (strain given by relative change in distance between movable walls), inducing aspect ratios of 2.5 (17.5% strain), 2.9 (22.5% strain) and 3.3 (27.5%) (compare Fig 3G). *Top row*: No MET was allowed after the pull. This was due to our initial hypothesis that somite doubling is nothing more than the reorganization of existing epithelial cells. We applied different strain values to see if high strain might lead to somite doubling. No somite division was observed. *Middle row*: MET was allowed after the pull if a cell had been in contact to the surrounding ECM matrix for a certain period of time. This MET rule did not allow somite division under various strain conditions. *Bottom row*: A new rule allows MET of mesenchymal cells upon contact to the lateral or basal membranes of epithelial cells. Formation of stable daughter somites could be observed.

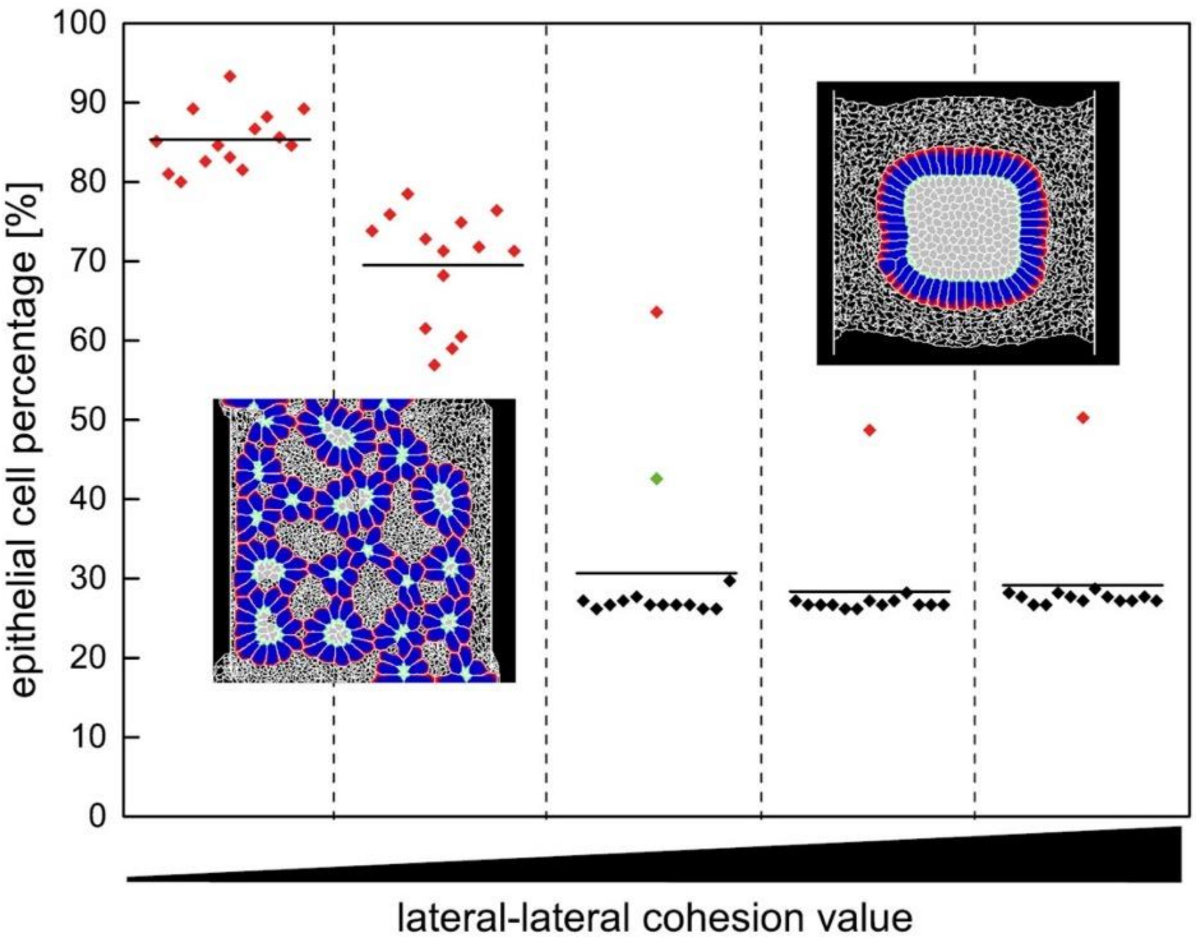

Fig S7: Influence of cohesion between lateral domains of epithelial cells on daughter somite number and epithelial cell percentages *in silico*, related to Figure 5. Cohesion between lateral domains of epithelial cells was varied in non-stretched somites to study the effect on somite formation. With low cohesion, almost 85% of mesenchymal cells became epithelial. This increase in epithelial cell number resulted in the formation of many epithelialized cell clusters (red data points), whereas for high cohesion values, we observed no somite division except for one case (green data point). Non-dividing somites are indicated by black data points.

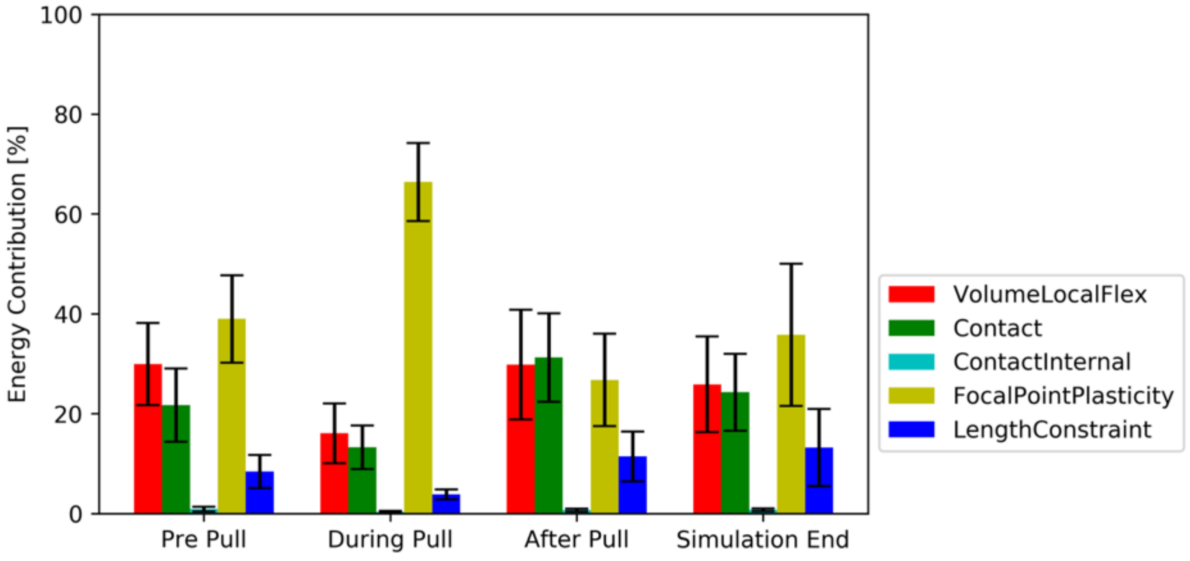

Fig S8: Contribution of the energy terms to the Hamiltonian, related to Figure 5. Terms are referred to by CompuCell3D plugin names; these are the software elements responsible the terms. The volume constraint (Eq. 3) restricts variations in the cell volume (VolumeLocalFlex). The contact energies (Eq. 2) are separated out for intercellular adhesion energy (Contact), i.e., contribution of cell-cell and cell-ECM adhesion and intracellular adhesion energy (ContactInternal), i.e., the adhesion between the compartments of the epithelial cells. The major contribution comes from the elasticity term (Eq. 4; FocalPointPlasticity). The elasticity term is responsible for the springs between the ECM volume elements and the springs between the compartments of the epithelial cells. The LengthConstraint plugin imposes elongation of a cell along an axis and ensures that cell elongation along the axis is closer to the target length. Shown are averages over n=14 simulations; error bars represent the 95% confidence interval.

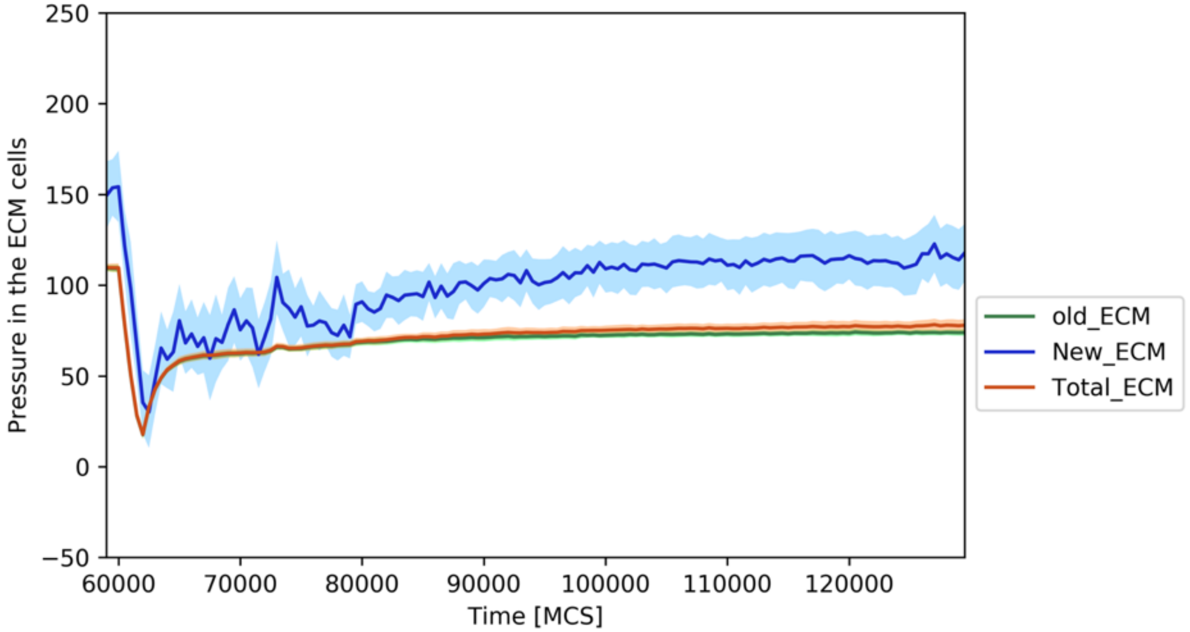

Fig S9: Pressure changes in the ECM cells over time, related to Figure 5. This plot shows pressure changes for old, new and total ECM during the simulation averaged over all cells in the simulation. At 60,000 MCS, axial outward pull is started. This pull brings the system under tension and causes a drop in the ECM pressure. After the pull, the ECM cells take some time to recover their pressure. However, only the newly formed ECM from the basal membranes of epithelial cells seems to have higher pressure. The newly formed ECM is the one which is secreted between the daughter somites whereas the old ECM is mainly on the outside. The dark lines show the average pressure over 14 simulations whereas slightly dull shaded area shows 95% confidence interval.

Supplemental tables

Table S1: Parameter values used in the Cellular Potts Model, related to Figure 5. Parameter values are given in simulation units and in the corresponding physical units, followed by a brief motivation. Energy (H) in the cellular Potts model relates to (cellular, adhesive, etc.) forces as $\vec{F} = \vec{\nabla}H$. Thus, estimates for the scaling of contact and elasticity energies to physical units can be based on measurements of the magnitude of the forces applied by the cells, the frequency by which pseudopods are extended and retracted ("Temperature"), the adhesion strengths between cells (see e.g., (Krieg et al., 2008)) and between cells and medium, combined with contact area, etc.

Parameter	Value in simulation units	Physical units	Motivation
Lattice spacing (Δx)	1 pixel width	0.5 µm	Chosen
Timestep	1 Monte Carlo Step (MCS)	0.36 s	Chosen
Lattice size	400 x 300 pixels	200 µm x 150 µm	Chosen
Cellular temperature	~100 energy unit (eu) per site update (i.e., 100 x lattice size eu / MCS = 12 x 10^6 eu/MCS)	-	Chosen; it reflects the rate of pseudopod extension and retraction and the forces that are applied
Somite diameter	~ 162 pixel widths	~ 80 µm	Somite is half the size of the *in vivo* somites (~160 µm) for simulation efficiency. The results are independent of simulation size
Total simulation steps	130,000 MCS	13:00 hours	Based on experiment
Start of pull	60,000 MCS	6:00 hours	Stabilisation of initial condition
Duration of pull	10,000 MCS	1:00 hours	Based on duration of single experimental pull
Epithelial target area V_{epi}	300 pixels (9% Apical, 70% Lateral and 21% Basal)	~ 75 µm^2	Measured using ImageJ from Kulesa and Fraser *Science* 2002 and Martins et al. PLOS ONE 2009[1]
Epithelial stiffness $\lambda_{volume-\ epi}$	100 energy unit (eu) per pixel^2	- (see Table caption regarding energy units)	Estimated based on experimental tissue organization
Mesenchymal target area V_{mes}	195 pixels	~50 µm^2	Measured using ImageJ from Martins et al. PLOS ONE 2009
Mesenchymal stiffness $\lambda_{volume-\ mes}$	30 eu/pixel^2		Estimated based on experimental tissue organization
ECM "cell" area	75 pixels	~20 µm^2	Chosen
ECM stiffness $\lambda_{volume-\ ecm}$	25 eu/pixel^2		Estimated based on experimental tissue organization
Epithelial polarization time	1500 MCS	~10 minutes	It takes 1 to 2 10-min t-frames in Video S3 of Martins et al. PLOS ONE 2009
Epithelial elongation time	1500 MCS	~10 minutes	*idem*
Epithelial sub-cellular spring stiffness	500 energy units per pixel width (eu/pw)	-	Estimated to approximate shape of epithelial cell

Apical-lateral spring length	15 pixel widths	7.5 µm	Estimated to approximate shape of epithelial cell
Apical – Basal spring length	30 pixel widths	15 µm	*As above*
Apical – Apical spring stiffness	100 energy units per pixel width (eu/pw)		*As above*
Apical – apical spring length	4 pixel widths	2 µm	*As above*
ECM-ECM spring stiffness	200 eu/pw	-	*As above*
ECM-ECM spring length	10 pixel widths	5 µm	*As above*
**Contact energies**			
Intercellular adhesion energies			
$J_{med-apical}$, $J_{med-lateral}$, $J_{med-basal}$, and $J_{med-mes}$	300 energy units per pixel width (eu/pw)	-	Estimated based on observed tissue organization, followed by sensitivity analysis (Fig. S7)
$J_{med-ecm}$	30 eu/pw	-	*As above*
$J_{apical-apical}$	3 eu/pw	-	*As above*
$J_{apical-lateral}$ and $J_{apical-basal}$	150 eu/pw	-	*As above*
$J_{apical-mes}$	160 eu/pw	-	*As above*
$J_{lateral-lateral}$	70 eu/pw	-	*As above*
$J_{lateral-basal}$	100 eu/pw	-	*As above*
$J_{lateral-mes}$	200 eu/pw	-	*As above*
$J_{lateral-ecm}$	130 eu/pw	-	*As above*
$J_{basal-basal}$	30 eu/pw	-	*As above*
$J_{basal-mes}$	280 eu/pw	-	*As above*
$J_{basal-ecm}$	40 eu/pw	-	*As above*
Internal adhesion energies between epithelial domains			
$J_{apical-lateral}$ and $J_{lateral-basal}$	2 eu/pw	-	*As above*
$J_{lateral-basal}$	20 eu/pw	-	*As above*

Transparent methods

Experimental Design

The objective of the study was to assess a possible role for mechanical strain in defining formation rate and total number of somites in a chick embryo. To that end we built an *ex ovo* embryo stretching device on top of our submerged filter paper sandwiches model and subjected young chick embryos to surplus axial tension (Fig 1). Deformations and possible changes to chick embryo morphology were recorded on-line by time-lapse imaging. To address specific questions, detailed imaging, immunohistochemistry and *in situ* hybridizations were performed after fixing of the embryos.

Embryo preparation and culture medium

Fertilized chicken eggs, white-leghorn, *Gallus gallus domesticus* (Linnaeus, 1758), were obtained from Drost B.V. (Loosdrecht, The Netherlands), incubated at 37,5°C in a moist atmosphere and automatically turned every hour. After incubation for approx. 33 h, HH8-9 chicken embryos were explanted using filter paper carriers (Chapman et al., 2001) and cultured *ex ovo* as modified submerged filter paper sandwiches (Fig 1A) (Chapman et al., 2001; Schmitz et al., 2016). Embryo culture medium consisted of Pannett-Compton (PC) saline (Pannett and Compton, 1924; Schmitz et al., 2016) mixed with freshly harvested thin albumen in a 3:2 ratio. PC stock solutions can be stored at 4 ºC for several months, but PC saline (mixture of stock solutions and MilliQ-water) should be prepared freshly every week and stored at 4ºC between experiments. Addition of Penicillin/Streptomycin (10000 U/ml) in 100x dilution prevents occasionally appearing bacterial infections. Silicone sheets further protected embryos in culture from infections as well as from convection of the medium. For the production of the silicon sheets, see Transparent Methods. Filter paper carriers were cut from thick filtration paper with a laser cutter according to the dimensions depicted in Figure 1D (Schmitz et al., 2016). Four holes were cut out from corners of the carriers (Fig 1D) to hook the filter paper sandwiches onto the pins of the motorized arms of the stretching setup (Fig 1A7, 1C).

Experimental setup - Embryo stretcher

Embryos were cultured and mechanically manipulated on a custom-made embryo stretcher (Fig 1). This setup allows to culture up to three embryos simultaneously in a variant of the earlier described “submerged filter paper sandwich” (Schmitz et al., 2016). The setup consists of a temperature-controlled medium container surrounded by a metal frame, which carries two motorized translation stages mounted on opposing sides. The stages were controlled by a custom-made LabVIEW routine, which allowed defining tension profiles for overnight experiments (see ‘Stretching protocol’ below). Embryos were fully submerged in the culture medium described above and the setup was prepared for an experiment as follows: The temperature-controlled beaker was placed in the centre of the motorized x-y-stage of the upright zoom microscope. Then the temperature-controlled beaker was filled with 200 mL of clean culture medium. The temperature of the beaker was set to 40°C and the silicone sheets were placed into the setup. Air bubbles were removed with a plastic transfer pipette. Then a chick embryo was explanted into a filter paper sandwich (Schmitz et al., 2016) (Fig 1A) and immediately submerged into the culture medium. After clamping three filter paper sandwiches into the setup, each filter sample was cut perpendicularly to the embryonic axis, about 1 mm posteriorly of the widest point of the elliptical aperture (dashed red line in Fig 1A-9). Then the culture medium was covered with 50 mL of light mineral oil using a plastic transfer pipette (Fig 1A-10).

Stretching protocol, axial deformation and somite deformation

Embryos were exposed to a standardized stretching protocol in the embryo stretcher (Fig 1C). In our original protocol, chick embryos were elongated at continuous speeds ranging from slow (1.3 µm/min, roughly matching the embryo’s natural elongation speed) to fast (8 µm/min). It was at 8 µm/min and after approx. 16 hours of stretching that we first observed the division of somites. However, embryos frequently ruptured, thus inhibiting a robust and repeatable observation of the phenomenon. In order to better study the dividing somites, we decided to take a different approach: We increased the stretching speed to 1.2 µm/s and applied the desired deformation during two relatively short stretching intervals (51 to 55 min) separated by a resting interval of two hours to allow damaged tissue to heal. During each stretching interval the displacement of the computer-controlled metal arm (see red arrow in Fig 1) extended the filter paper sandwiches by 3.8 mm at a speed of 1.2 µm/s. Subsequently, we calculated the resulting mechanical strain of the embryos for the first and the second stretching interval as relative length change compared to the axial length of the embryos before stretching. The first stretch led to 23 ± 3 % strain (average and standard deviation over 57 embryos); the second pull caused 19 ± 3 % strain.

Average somite formation period

To assess the hypothesized influence of mechanical tension on somitogenesis, we determined the average somite formation period for stretched and control embryos using our dark field microscopic time-lapse movies. We counted the number of somites in stretched embryos at the end of the second pull and at the end of the experiment. If a somite had not completely separated from the PSM at the end of the second pull, the counting was started after formation of the following somite. From the total number of somites formed after the application of the second pull and the corresponding time interval we calculated the average somite formation period. The somite formation period for control embryos was determined accordingly from the beginning of the culturing in the submerged filter paper sandwich.

Immunohistochemistry

After the pulling experiments, the embryos and age-matched controls were fixed in 4% paraformaldehyde overnight in PBS at 4_{o}C. Permeabilization in PBST + 0.15% Triton-X-100 lasted for 1.5 hours. Blocking was performed for 2 hours in PBST + 2% BSA + 5% normal goat serum. The following antibody was used: fibronectin mouse-anti-chicken (B3/D6-s, Hybridoma bank). The antibody was diluted in PBST with 1% BSA. Embryos were incubated in primary antibody solution for 24h at 4_{o}C, followed by extensive washing in PBS and incubation with appropriate Alexa Fluor-conjugated secondary antibody (1:500, Molecular Probes). Embryos were stained for F-actin using Alexa Fluor 546 Phalloidin (1:200, Molecular Probes) and for nucleic DNA using DAPI (1 µg/ml). Cell proliferation and apoptosis staining was performed using following antibodies: rabbit polyclonal anti-cleaved caspase-3 (1:200, Cell Signaling) and rabbit polyclonal anti-phosphohistone-H3 (1:400, Cell Signaling) with the appropriate Alexa Fluor-conjugated secondary antibodies (1:500, Invitrogen) and DAPI for nucleic DNA (1 µg/ml).

Proliferation rate and apoptotic rate

Apoptotic (Cas3 staining, control n=4, pulled n=4 embryos) and proliferating (pHH3 staining, control n=6, pulled n=6 embryos) cells in somitic mesoderm lanes (the newest somites I to VI) were counted in high-resolution confocal micrographs acquired on a Leica SP8 confocal microscope. At least 500 cells were counted per embryo. Apoptotic rate and proliferation rate were calculated as follows: (apoptotic/proliferation) rate (%) = number of positive staining cells/number of total cells×100.

Epithelial cell percentages

The percentage of epithelial cells in the equatorial z-plane of 13 daughter somite pairs, originating from the same mother somite, and 22 control somites was determined (*in vivo)*. To that end, high-resolution confocal micrographs of embryos stained with DAPI for nucleic DNA were acquired on a Leica SP8 confocal microscope. Then somites were counted for (mesenchymal) core cells and epithelial cells to calculate epithelial cell percentages. *In silico,* cell percentages in 12 daughter somite pairs and 12 control somites were counted accordingly.

Aspect ratio determination and ROC curve

The geometry of *in vivo* somites in controls and stretched embryos was described by measuring their length in rostro-caudal (x) and their width in medio-lateral (y) direction using the “Measure”-tool in ImageJ. Subsequently, the corresponding aspect ratio AR (AR = x/y) was calculated. Somites forming in controls and in stretched embryos after the second pull were measured upon their separation from the anterior tip of the PSM. Somites that had been formed before were measured at the end of the second pull. The aspect ratio of *in silico* somites was determined before and after the application of the pull accordingly (for strain regimes *in silico* see Cellular Potts model below).

Somite and somitocoel volume measurements

Z-stacks of somites V or VI in control and pulled embryos were acquired on a Leica SP8 confocal microscope. Stacks were obtained with 1 µm steps in z, with enough ventro-dorsal distance for imaging the whole mesoderm. The volumes of the whole somite or the somitocoel were compared between three groups: control, pulled somites that had not divided, and pulled somites that had divided (Fig 5I and 5J). To compare the somite and somitocoel volumes, the volumes were measured in fixed embryos. Confocal images were processed and converted with ImageJ (https://imagej.nih.gov/ij/). Manual segmentation, 3D reconstruction and volume analysis were then performed with 3D Slicer (https://discourse.slicer.org/), via the Segment Editor for segmentation, and Segment Statistics to calculate the volume. For the volumes of the divided somites/somitocoels, the volumes of both daughter somites were summed together. For statistical analysis, unpaired t-tests with Welch’s correction for standard deviation were performed with Graphpad.

In situ hybridizations

In situ hybridizations were performed by standard procedures. Embryos were fixed in freshly prepared 4% PFA in PBS. The embryos were pre-treated with proteinase-K in PBST at 37°C with agitation for 3 minutes. During staining, embryos were incubated in NTMT containing 4.5 µl NBT (75mg/ml in 70% DMF) and 3.5 µl BCIP (50mg/ml in 100% DMF) per 1.5 ml. Pulled embryos and age-matched controls were stained in the same wells for the same time, as much as possible. After the staining had been stopped, the embryos were photographed in glycerol 80% in H_2O with a Leica DFC320 camera on a Leica MZ75 microscope. Due to the relaxation of the stretched embryos, after their release from the embryo stretcher, the embryos tend to roll up. In order to photograph these embryos, they were placed on agar and below Corning cover glasses X2000 #1 (Fisher Scientific), with spacers and weights to hold them flat. This contributed to partly varying photo conditions for the in situ hybridization panels Fig 4H-M.

Cellular Potts model of somite division

To develop new hypotheses for the mechanisms underlying the somite division observed *in vivo*, we constructed a two-dimensional mathematical model based on the Cellular Potts Model (Glazier and Graner, 1993; Graner and Glazier, 1992), representing a cross-section through a three-dimensional somitic tissue. The model simulations were based on a Cellular Potts Model (CPM), also known as Glazier-Graner-Hogeweg model (Graner and Glazier, 1992). The model was implemented using CompuCell3D, an open source modelling package based on the CPM (Dias et al., 2014). The mesenchymal cells were modelled as regular cellular Potts cells, whereas for the epithelial cells we used a compartmental CPM (Dias et al., 2014), which represents biological cells as a collection of sub-cellular domains. The extracellular matrix was modelled as a collection of small volume elements connected to one another and to the epithelial cells by Hookean springs. Our compartmentalized CPM projects biological cells on a regular, square lattice as domains of (usually) connected lattice sites. Each lattice site, $\vec{x}$, corresponds to a cross-sectional area of approximately 0.5 µm × 0.5 µm and is associated with a domain index $\sigma(\vec{x}) \in \mathbb{Z}^{0,+}$ that uniquely identifies a whole cell, a cellular compartment, or a volume element of extracellular material. Cell identification number $\sigma = 0$ represents a generic 'medium'. Each domain σ has a type label $\tau(\sigma) \in \mathbb{N}$ to represent the generic 'type' (subcellular domain, ECM, and so forth) and an additional label $\xi(\sigma(\vec{x})) \in \mathbb{N}$ that bundles compartments to a biological cell or connected extracellular material. Although each individual object (subcellular compartment, ECM medium etc.) has its own unique domain index σ, many objects may be associated with the same type label τ, and many objects of the same of different type τ may form one biological object (e.g., an epithelial cell) with label ξ.

The evolution of our CPM is governed by a force-balance represented by Hamiltonian H:

$$H = H_{contact} + H_{volume} + H_{spring} \ , \qquad (Eq. 1)$$

which governs the dynamics of cells (e.g. cell behaviours, properties and interactions). Ignoring cell inertia, from this Hamiltonian the forces are recovered as $\vec{F} \propto \vec{\nabla} H$. The Hamiltonian is minimized using a Metropolis algorithm that mimics microscopic membrane and material fluctuations, such that both the equilibrium and the transient towards the equilibrium can be physically and biologically interpreted. $H_{contact}$ represents cell adhesion where cell-cell and cell-medium interactions take place through contact energies. The length of the interface between two cells defines the contact energy and is given by:

$$H_{contact} = \sum_{(\vec{x},\vec{x}')} J_{\tau(\sigma(\vec{x})),\tau(\sigma(\vec{x}'))} \left(1 - \delta_{\sigma(\vec{x}),\sigma(\vec{x}')}\right) \qquad (Eq. 2)$$

Here, $J_{\tau(\sigma(\vec{x})),\tau(\sigma(\vec{x}'))}$ is the bond energy between two neighbouring cell types $\tau(\sigma(\vec{x}))$ and $\tau(\sigma(\vec{x}'))$, and $\delta_{\sigma(\vec{x}),\sigma(\vec{x}')}$ is the Kronecker delta term in which adhesion is restricted to the cell membranes by eliminating the contribution from the neighbouring lattice sites belonging to the same cell. If $\sigma(\vec{x}) = \sigma(\vec{x}')$, the delta function returns a value of 1 and 0 otherwise. The term H_{volume} in the Hamiltonian specified in Eq. 1 is given by:

$$H_{volume} = \sum_{\sigma} \lambda_{volume}(\sigma).[v(\sigma) - V(\sigma)]^2 \qquad (Eq. 3)$$

and constrains the cell area, $v(\sigma)$, close to a resting area $V(\sigma)$. The Lagrange multiplier λ_{volume} represents cell elasticity - higher values of λ_{volume} reduce fluctuations of a cell's area from its target area.

Compartments of cells and subunits of the extracellular matrix can be mechanically coupled by Hookean springs that connect their centres of mass. Each spring contributes an additional energy bias H_{spring} to the Hamiltonian in Eq. S2, as

$$H_{spring} = \sum \lambda_{ij} \left(l_{ij} - L_{ij} \right)^2 \qquad (Eq.4)$$

where l_{ij} is the absolute distance between the center of mass of cells i and j, and L_{ij} is a resting spring length. $\lambda_{ij}$is an elasticity parameter. Springs rupture if they exceed a threshold length; new springs are formed if cells move within a threshold distance. In our simulations, we have many cell types (epithelial internal compartments, extracellular matrix (ECM), and epithelial cell (apical)) which are connected using springs of which the parameters vary per cell type as explained in the Transparent Methods.

The CPM is updated using a Metropolis algorithm, which mimics the extension and retraction of pseudopods of the biological cells, and fluctuations of the extracellular matrix materials. The algorithm iteratively takes a randomly chosen lattice site $\vec{x}$ and attempts to copy its cell index $\sigma(\vec{x})$ into a randomly chosen adjacent lattice site $\vec{x}'$. This is called a copy attempt. The probability of accepting or rejecting the attempted copy is based on the energy minimization criteria and follows the Boltzmann probability,

$$P\left(\sigma(\vec{x}) \rightarrow \vec{x}'\right) = \begin{cases} 1 & , \Delta H\left(\sigma(\vec{x}) \rightarrow \vec{x}'\right) < 0 \\ e^{\frac{-\Delta H(\sigma(\vec{x}) \rightarrow \vec{x}')}{T}} & , \Delta H\left(\sigma(\vec{x}) \rightarrow \vec{x}'\right) \geq 0 \end{cases} \qquad (Eq.5)$$

where $\Delta H(\sigma(\vec{x}) \rightarrow \vec{x}')$ represents the change in the Hamiltonian due to the copy attempt. If the attempted copy update would reduce the energy, i.e. $\Delta H(\sigma(\vec{x}) \rightarrow \vec{x}') < 0$, the update is accepted with a probability of 1. If the energy increases due to the copy-attempt, the system follows Boltzmann probability to accept or reject a copy-attempt. The parameter T is the cellular temperature, representing the amplitude of active cell membrane fluctuations or fluctuations of the extracellular materials. Simulation time proceeds in *Monte Carlo Steps* (MCS); One MCS corresponds to 0.36 seconds of experimental time, and consists of *N* copy attempts, with *N* the number sites in the lattice. All parameters are given in dimensionless units in Table S1 alongside interpretation in terms of physical units and a brief motivation for the values used. Further model assumptions, a detailed description of the somite stretching protocol, a study of the relative contributions of the mechanisms represented by the Hamiltonian, and a parameter sensitivity analysis are in the Supplemental Materials.

Statistical Analyses

For assessing epithelial cell percentage in the somites, statistical analysis was performed using GraphPad Prism software. Unpaired parametric two tailed t-tests (with Welch's correction for unequal variance) were applied to determine P-values for the epithelial percentages shown in the graph in Fig 3E. The percentages of epithelial cells change significantly *in vivo* (P<0.0001) and *in silico* (P<0.0001).
Statistical analyses of the proliferation and apoptosis rates were performed using GraphPad Prism software. Mann–Whitney unpaired non-parametric two-tail testing was applied to determine the P-values for the apoptotic and proliferation rates, respectively. shown in Fig 5F.
The receiver operating characteristics (ROC) curves (Hanley and McNeil, 1982) (Fig 3H) were generated by performing a binary logistic regression using the Data Analysis Tool of the Real Statistics Excel plugin Realstats (available at http://www.real-statistics.com). We analysed how well the aspect ratio of stretched somites *in vivo* and *in silico* can predict the binary outcome of whether a somite will undergo division or not. This is measured by the area under the curve (AUC) in the ROC diagram. The AUC can vary between 0.5 (stochastic relation) and 1 (fully determined). 95% Confidence intervals for AUC values were calculated using the ‘ROC curve analysis’ tool of MedCalc software (available at https://www.medcalc.org/index.php).

Silicone sheets

Silicone sheets protected embryos in culture from convection of the medium, thereby avoiding additional damage. Silicone sheets (ca. 350 µm in thickness) were made using a Sylgard® 184 Silicone Elastomer Kit as follows: A 15-cm plastic petri dish was placed on a scale and 6.165 g (5.554 mL) of base solution were pipetted into its centre using a plastic transfer pipette (cut off tip). Then 0.206 g (0.2 mL) of curing agent were added using a glass pipette. Base and curing agent were mixed slowly, using a wooden spatula and spread out over the bottom of the petri dish. The petri dish was placed into a vacuum chamber for 2 hrs to remove air bubbles and let silicone solution spread out equally. Exposure to 80 °C for ca. 2 hrs let silicone polymerize and cure. Afterwards, silicone was let to cool to room temperature for about 5 hrs or overnight. Tweezers were used to free the borders of the silicone sheet from the walls of the petri dish and peel the sheet from the culture dish (wear gloves). Silicone was stored between sheets of a plastic document sleeve to prevent accumulation of dust. For preparing silicone sheets fitting in the setup, the plastic sleeve was removed from one side of the silicone sheet and the plastic stencil (Fig 6D) placed on it. A razor blade was used to cut around the outline of the stencil and the ten holes indicated by the stencil were cut out using a

hole puncher. After removing the other plastic sleeve layer, silicone sheets were stored in a closed 10 cm petri dish.

Cellular Potts Model assumptions

The model included the following assumptions: (i) the tissue surrounding the somite can be approximated as elastic, and was modelled as a non-specified extracellular matrix (ECM); (ii) the somite consists of polarized epithelial cells forming the outer layer (Dias et al., 2014), and the somite core (somitocoel) consists of unpolarized mesenchymal cells. The mesenchymal cells in the core of the somite were represented by single-compartment, non-coupled and non-polarized cells. Following Dias *et al.* (Dias et al., 2014) the epithelial cells in our model consisted of three domains, called 'apical', and 'lateral' and 'basal' (Fig S5). The three compartments were initially distributed at random inside an epithelial cell and after a brief relaxation period of 1500 MCS (epithelial polarization time), these compartments were connected internally to one another using linear elastic springs (Eq. 4). To achieve epithelial elongation, target lengths of all internal springs ($L_{apical-lateral}$, $L_{apical-basal}$, $L_{lateral-basal}$) were individually incremented by 1 every 20th MCS within the elongation time frame (1500 MCS, approximately 9 min), until every spring reached its final specified target length ($L_{apical-lateral} = 15, L_{apical-basal} = 30,\ L_{lateral-basal} = 20$). We have used springs between adjacent cells (such as apical compartments of epithelial cells or ECM cells) to represent strong Cadherin or tight junctions. Within one epithelial cell, springs are used to make epithelial cells elongated and to have domains with different adhesion properties. These assumptions have been adapted from (Dias et al., 2014). The ECM, with its main functional component fibronectin *in vivo* (Rifes and Thorsteinsdóttir, 2012), was modelled as a network of compartments connected by Hookean springs of resting length $L_{ecm-ecm} = 10$ with elastic stiffness $\lambda_{ecm-ecm} = 200$. These parameters were chosen such that the ECM stays intact during and after stretching.

We first attempted to construct a well-organized, initial epithelial structure as a starting point for the stretching model. Contact energies between domains as well as contact energies with other cell types and the ECM were set according to Table S1. In absence of quantitative values for the adhesion strengths and interfacial tensions between the cells, we estimated parameter values for which a stable epithelial monolayer is maintained in our simulations, followed by parameter sensitivity studies. We assumed the apical domains of adjacent epithelial cells cohered strongly, following Dias *et al.*'s assumption mimicking the distribution of N-Cadherin *in vivo* (Dias et al., 2014). The lateral domains of epithelial cells (between the apical and basal domains) adhere strongly to each other, similar to Cadherin mediated cohesion *in vivo* (Horikawa et al., 1999). To represent the apical actin ring, each centre of mass of an apical unit was connected to the centre of mass of neighbouring apical domains on either side (left and right) using elastic springs of a resting length of $L_{ij} = 4$ with elastic stiffness $\lambda_{apical-apical} = 100$.

The monolayer of epithelial cells was constructed by initializing the simulation with a collection of mesenchymal cells surrounded by an elastic ECM. We selected a mesenchymal cell at the boundary with the surrounding ECM and made it epithelial. This first epithelialized cell induced MET in neighbouring cells based on basolateral contact (Jackson et al., 2017) where MET was implemented by turning over a mesenchymal cell into a compartmentalized, unpolarized epithelial cell. Subsequent MET of the adjacent mesenchymal cells finally led to a fully epithelialized somite-like structure. After a stable, somite-like, epithelial structure had formed, we gradually strained the extracellular matrix in our simulations, in order to mimic the experimental setup. To this end, we connected two 'walls' constructed out of immobile cells to the left and right-hand ends of the ECM using stiff elastic springs and slowly moved the walls apart by 1 pixel every 50 MCS (~ 1.7µm/min), similar to the compression of tissue spheroids (Marmottant et al., 2009)and the application of stents in arteries (Tahir et al., 2015).

Somite stretching in silico

The stretching rate was sufficiently slow (walls moved outward by 1 pixel every 50 MCS, corresponding to ~ 1.7µm/min), such that it did not damage the ECM cells (Movie S3). Upon stretching, several springs between neighboring apical compartments released and mesenchymal cells from the core became exposed to the lateral or basal membranes of epithelial cells leading to additional MET. These additional epithelial cells disturbed the equilibrium and could not get incorporated into the original epithelial ring. So, the epithelium started to reorganize and divide into daughter somites (Fig S6, bottom row and Movie S3). To systematically analyse how well the geometry of stretched somite predicts division we increased the distance between the lateral walls slowly by 30 to 110 pixels (corresponding to 15 µm to 55 µm), resulting into aspect ratio values similar to stretched somites *in vivo* (Fig 5G).

Fibronectin deposition

Based on our observations that daughter somites are separated and presumably stabilized by a newly forming fibronectin matrix (Fig 3), we implemented a similar rule for ECM production by the basal units of epithelial cells. If the basal domain of an epithelial cell is not attached to a specified amount of ECM (given by threshold value) for a certain duration, it produces an additional ECM cell. This production continues until the threshold value is reached again. Such production of the fibronectin allows the dividing somites to separate from each other permanently.

Parameters
The parameters used in the simulations are listed in Table S1, alongside their interpretation in terms of physical units and a brief motivation.

Model validation
The *in silico* somite model can be parameterized to the experimentally observed ratio of mesenchymal and epithelial cells**.** Following the MET based on the basolateral contact rule, we observed that initial epithelialization of the somite and division of the somites after stretching can be achieved both with a large core and small core somite. To further validate the *in silico* model, we also tested the influence of decreased cohesion between lateral domains of epithelial cells in epithelializing, non-stretched somites. Similar to results in *N-Cadherin/cad11* double-homozygous mouse mutants (Horikawa et al., 1999), we observed subdivisions into small cell clusters of epithelioid morphology (Fig S7).

Energy
We also tested the relative contribution of each energy term in time at different stages in the simulation (Fig. S8). The elasticity terms (Eq. 4) have a major contribution towards the overall system energy. This is responsible for all the inter- and intracellular springs in the model. The contribution of elasticity is substantially higher during the pull and also higher during pre-pull and at the end of simulations.

Pressure changes
From Fig S7, one can see small ECM cells between the daughter somites. This suggests a slightly higher pressure in middle of the daughter somites, which could be due to the production of the new ECM cells from the basal membranes of epithelial cells. In order to evaluate the pressure changes in the ECM cells, we estimated the pressure in the ECM cells over time using:

$$\wp = 2\lambda(\mathrm{V_i} - \mathrm{v}_\sigma)$$

where $\mathrm{V_i}$ is the target volume and v_σ is the current cell volume. λ is an inelasticity constant. Fig S9 shows that axial pull initially causes a drop in the ECM cell pressure, but after some time the pressure was regained. The newly formed ECM cells initially have a higher pressure than the old ECM cells. This is because the whole system is continuously under tension and ECM acts like a spring mesh. Since there is no empty space for new cells inside the ECM mesh, it takes time for new ECM cells to achieve or maintain their volume. For this reason, new ECM cells are under slightly more pressure than the old ECM cells.

Supplemental References

Chapman, S.C., Collignon, J., Schoenwolf, G.C., Lumsden, A., 2001. Improved method for chick whole-embryo culture using a filter paper carrier. Dev. Dyn. 220, 284–289. https://doi.org/10.1002/1097-0177(20010301)220:3<284::AID-DVDY1102>3.0.CO;2-5

Dias, A.S., de Almeida, I., Belmonte, J.M., Glazier, J.A., Stern, C.D., 2014. Somites Without a Clock. Science (80-.). 343, 791–795. https://doi.org/10.1126/science.1247575

Glazier, Graner, 1993. Simulation of the differential adhesion driven rearrangement of biological cells. Phys. Rev. E. Stat. Phys. Plasmas. Fluids. Relat. Interdiscip. Topics 47, 2128–2154.

Graner, F., Glazier, J.A., 1992. Simulation of Biological Cell Sorting Using a 2-Dimensional Extended Potts-Model. Phys. Rev. Lett. 69, 2013–2016. https://doi.org/10.1103/PhysRevLett.69.2013

Hanley, A.J., McNeil, J.B., 1982. The Meaning and Use of the Area under a Receiver Operating Characteristic (ROC) Curve. Radiology 143, 29–36. https://doi.org/10.1148/radiology.143.1.7063747

Horikawa, K., Radice, G., Takeichi, M., Chisaka, O., 1999. Adhesive Subdivisions Intrinsic to the Epithelial Somites. Situ 215, 182–189. https://doi.org/10.1006/dbio.1999.9463

Jackson, T.R., Kim, H.Y., Balakrishnan, U.L., Stuckenholz, C., Davidson, L.A., 2017. Spatiotemporally Controlled Mechanical Cues Drive Progenitor Mesenchymal-to-Epithelial Transition Enabling Proper Heart Formation and Function. Curr. Biol. 27, 1326–1335. https://doi.org/10.1016/j.cub.2017.03.065

Krieg, M., Arboleda-Estudillo, Y., Puech, P.H., Käfer, J., Graner, F., Müller, D.J., Heisenberg, C.P., 2008. Tensile forces govern germ-layer organization in zebrafish. Nat. Cell Biol. 10, 429–436. https://doi.org/10.1038/ncb1705

Marmottant, P., Mgharbel, A., Kafer, J., Audren, B., Rieu, J.-P., Vial, J.-C., van der Sanden, B., Maree, A.F.M., Graner, F., Delanoe-Ayari, H., 2009. The role of fluctuations and stress on the effective viscosity of cell aggregates. Proc. Natl. Acad. Sci. U. S. A. 106, 17271–17275. https://doi.org/10.1073/pnas.0902085106

Pannett, C., Compton, A., 1924. the Cultivation of Tissues in Saline Embryonic Juice. Lancet 203, 381–384. https://doi.org/10.1016/S0140-6736(01)15954-4

Rifes, P., Thorsteinsdóttir, S., 2012. Extracellular matrix assembly and 3D organization during paraxial mesoderm development in the chick embryo. Dev. Biol. 368, 370–81. https://doi.org/10.1016/j.ydbio.2012.06.003

Schmitz, M., Nelemans, B., Smit, T.H., 2016. A submerged filter paper sandwich for long-term ex ovo time-lapse imaging of early chick embryos. J. Vis. Exp. 54636. https://doi.org/10.3791/54636

Tahir, H., Niculescu, I., Bona-Casas, C., Merks, R.M.H., Hoekstra, A.G., 2015. An in silico study on the role of smooth muscle cell migration in neointimal formation after coronary stenting. J. R. Soc. Interface 12, 20150358.
